# Imaging‐Guided Live Single‐Cell Lipid Profiling of Leader and Follower Cells During Collective Migration of Triple‐Negative Breast Cancer Cells

**DOI:** 10.1002/advs.75862

**Published:** 2026-06-16

**Authors:** Xiaoyue Huang, Judit González Riera, Mai Nguyen, Jeremy Broekhuis, Sylvia E. Le Dévédec, Ahmed Ali, Thomas Hankemeier

**Affiliations:** ^1^ Metabolomics and Analytics Centre Leiden Academic Centre For Drug Research Leiden University Leiden The Netherlands; ^2^ Institute of Biology Leiden University Leiden The Netherlands; ^3^ Division of Cell Systems and Drug Safety, Leiden Academic Centre For Drug Research Leiden University Leiden The Netherlands

**Keywords:** collective migration, direct infusion, lipid profiling, live single‐cell, nano‐ESI, TNBC

## Abstract

Leader and follower cells drive collective cancer cell migration through distinct and interactive roles, but their specific metabolic signatures remain largely unknown. Here, we developed and applied a workflow that integrates time‐lapse imaging, single‐cell tracking, microsampling and direct infusion high‐resolution mass spectrometry to profile the lipidomes of individually sampled leader and follower cells from highly migratory TNBC lines HCC1143 and HCC38. Approximately 120 metabolites were detected per cell at the MS^1^ level, and 70 compounds were assigned based on matched MS^2^ fragments, indicative of detected head‐group or side‐chain fragment ions. Distinct leader‐follower differences emerged in each cell line. HCC38 cells showed increased fatty acids (FA) in leader cells. In addition, alterations in several phosphatidylethanolamine (PE) and diacylglycerol (DG) species between leader and follower cell subpopulations were detected. Alternatively, leader and follower cells in HCC1143 exhibited changes in phosphatidylcholine (PC) species, with PC(34:1) confirmed by a fragment ions at m/z 184.0733. Overall, this study revealed the dynamic lipid metabolism in live migratory cancer cells, and highlights the potential of integrated dynamic imaging and single‐cell mass spectrometry for studying functional heterogeneity within leader‐follower cancer phenotypes.

## Graphical abstract

Integrated single‐cell mass spectrometry reveals distinct lipidomic signatures that differentiate leader and follower phenotypes in migratory breast cancer cells. Phenotype‐specific alterations across various lipid classes, including fatty acids and phosphatidylcholines, underpin functional heterogeneity. Uncovering these dynamic metabolic profiles provides crucial insights into collective cancer cell migration utilizing advanced live‐cell microsampling.

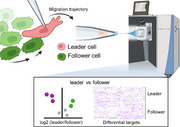

## 1 Introduction

Metastasis is responsible for approximately 90% of cancer‐related deaths [1]. Despite this, metastatic disease remains a major barrier to effective treatment. This is particularly true for highly resistant and heterogeneous tumors such as triple‐negative breast cancer (TNBC), which lacks common therapeutic targets and is associated with poor prognosis [2, 3]. The lack of effective treatment for metastasis in general, and for TNBC in particular, is complicated by our limited understanding of the cellular mechanisms driving the metastatic process [4]. Cancer cells disseminate from the primary tumor to distant organs through complex modes of migration, including single‐cell and collective migration [5], with the latter representing the predominant mode in most solid tumors [6]. Recent studies have identified specialized subpopulations of leader cells at the forefront of collectively migrating cancer cell groups [7]. In TNBC and other cancer types, these leader cells actively invade surrounding tissues, sense chemotactic gradients, and determine the overall direction of collective movement [8, 9, 10], while follower cells trail behind, relying on leader cells' cues for directional migration and contributing to tissue integrity [11]. Despite their critical role in the metastatic process, leader and follower cells remain understudied.

Recent efforts to dissect the differences between leader and follower cells have largely focused on genomic and phenotypic features across multiple cancer types. For example, Yamaguchi et al. showed that, in MDCK cells, leader cells display a distinct phenotype characterized by upregulation of Rac, integrin β1, and PI3K [12]. Furthermore, ablation of leader cells caused follower cells to stop migrating, underscoring the essential role of leader cells in collective migration. In a lung cancer spheroid model, an image‐guided genomics approach was used in which photoconverted cells at the spheroid front were isolated by fluorescence‐activated cell sorting (FACS); spheroids derived from leader cells exhibited greater invasive capacity than spheroids derived from follower or parental cells. Adding leader cells to follower spheroids further enhanced chain‐like invasion in both 3D and 2D assays. Transcriptome profiling revealed increased vascular endothelial growth factor (VEGF) transcripts in leader cells, which also showed higher vimentin and lower N‐cadherin expression compared with follower cells [13]. In another study, invasive cells leading strands in luminal breast cancer organoids were found to be molecularly and behaviorally distinct from the bulk tumor cells and expressed the basal epithelial genes cytokeratin‐14 and p63 [14]. Interestingly, these invasive leader cells did not express the canonical EMT markers Twist, Slug, or vimentin. Together, these studies indicate that, in addition to the morphological changes associated with leader cells, adhesion‐motility transitions and signaling pathways are critical regulators of collective migration. These processes are tightly coupled to alterations in energy metabolites and lipid profiles, which are key determinants of membrane dynamics, energy homeostasis, and signaling [15]. While some studies have reported differences in energy metabolites such as ATP and glucose between leader and follower cells [16, 17], lipidomic changes in leader cells remain largely unexplored, particularly in TNBC.

Current studies on lipid alterations in TNBC metastasis have largely relied on bulk measurements of cell subpopulations [10, 18]. Glycerophospholipids, the main structural lipids of eukaryotic membranes, are frequently reported as upregulated in TNBC. For example, comparative lipidomic profiling of metastatic versus primary TNBC tumors identified 71 lipid species that were increased and 70 that were decreased, with many upregulated lipids belonging to PC and PE species, including PC(16:0/20:4), PC(18:0/20:4), and PC(18:1/20:4) [19]. In another study, lipid rafts enriched in cholesterol and sphingolipids accumulated in the protrusions of migrating breast cancer cells [20], suggesting that similar lipid remodeling may occur in leader cells, which typically undergo pronounced morphological changes. Although these bulk‐level analyses demonstrate that specific lipid species are characteristic of metastatic TNBC and highlight their potential as biomarkers, they cannot resolve single‐cell heterogeneity or the functional lipid dynamics that distinguish leader from follower cells [21, 22, 23]. Single‐cell metabolomic approaches offer a way to overcome these limitations. For instance, polarity‐switching strategies have been shown to improve lipid annotation and coverage in single cells, supported by the development of high‐confidence in‐house lipid libraries and inter‐laboratory validation studies [24]. In addition, cross‐platform analyses have demonstrated the feasibility of single‐cell LC‐MS for lipid profiling, with hundreds of lipid species detected using nanoflow LC, polarity switching, and MS/MS acquisition on an Orbitrap Exploris 240 [25]. Structural lipidomics approaches, such as Paternò‐Büchi reaction coupled with tandem MS, further enable isomer‐resolved identification in single cells, including double‐bond localization and sn‐position assignment [26]. Despite these advances, limited MS/MS coverage often restricts metabolite confirmation and increases the risk of false positives in untargeted database matching, and high‐throughput platforms such as FACS‐based omics and microfluidic systems inherently disrupt the native microenvironment and alter the intrinsic metabolic state of adherent cells during detachment and sample preparation [27, 28].

To accurately capture the native‐state metabolome of these phenotypically‐defined leader and follower cells, a new platform is therefore needed that can sample cells in situ, preserve metabolic integrity, and provide sufficient sensitivity for high‐confidence structural identification. To address these gaps, we developed and applied a live single‐cell mass spectrometry workflow based on capillary microsampling combined with MS/MS acquisition and polarity switching. Notably, this approach enables, to our knowledge, the first integration of LC‐MS/MS‐based annotation with capillary‐sampled single‐cell lipidomic data. Using glass capillaries, live leader and follower cells from highly migratory TNBC cell lines (HCC38, HCC1143) were sampled under microscopic observation in their native microenvironment. Lipidomic profiling was then performed by nano‐electrospray ionization in both MS^1^ and MS/MS modes. By integrating time‐lapse imaging, live microsampling, and high‐resolution mass spectrometry, this workflow enables successful live single‐cell analysis during collective migration and supports MS^1^ and MS/MS‐based lipid identification. Defining these lipidomic differences between leader and follower cells may provide novel insight into the mechanisms that drive TNBC metastasis and reveal potential lipid‐related vulnerabilities for therapeutic interventions. This workflow provides a versatile foundation that can be further optimized for higher throughput and sensitivity, and readily extended to diverse biological contexts, underscoring its potential as a powerful tool for uncovering single‐cell lipidomic heterogeneity in a wide range of cellular processes and disease models.

## 2 Methods

### 2.1 Chemicals and Reagents

Organic solvents including methanol (MeOH), ethanol (EtOH), isopropyl alcohol (IPA), acetonitrile (ACN), and chloroform were LC‐MS grade (Biosolve Chimie, Dieuze, France). Water was LC‐MS grade and purchased from Thermo Fisher Scientific, the Netherlands. A lipid internal standard mixture, EquiSPLASH LIPIDOMIX (Avanti Polar Lipids, Alabaster, AL, USA, catalog No. 330731) was used as a multiclass internal standard. This mixture contains various lipid classes, including PC, lysophosphatidylcholine (LPC), PE, lysophosphatidylethanolamine (LPE), sphingomyelin (SM), ceramide (Cer), cholesterol ester (CE), diglyceride (DG), triglyceride (TG), phosphatidylinositol (PI), phosphatidylserine (PS), and phosphatidylglycerol (PG). An individual lipid standard, PC(15:0/15:0), was also purchased from Avanti Polar Lipids (Alabaster, AL, USA, catalog No. 850350).

### 2.2 Cell Culture

Five TNBC cell lines (SUM149PT, HCC1143, HCC1937, MDA‐MB‐231, and HCC38) were kindly provided by Prof. J. Martens from the Erasmus Medical Center. All cell lines were cultured in RPMI 1640 medium (Gibco, Life Technologies, USA, catalog No. 52400‐025) supplemented with 10% fetal bovine serum (FBS) and 1% penicillin‐streptomycin (Invitrogen). All cell lines were cultured at 37°C in an incubator with 5% CO_2_. The culture medium was replaced every three days, and cells were trypsinized at approximately 80% confluency using trypsin. Cell lines have tested negative for possible mycoplasma infection. Cell number was determined by trypan blue staining using an automated TC‐20 cell counter (Bio Rad, Hercules, California, USA, catalog No. 1450102). Cells were seeded into 35 mm culture dishes (ibidi GmbH, Germany, catalog No. 81156) and cultured for 48 h prior to analysis. Cells up to passage 20 were used in the experiments.

### 2.3 Wound‐Healing Assay

Cells were seeded at a density of approximately 2 × 10^5^ cells/mL into 35 mm culture dishes (ibidi GmbH, Germany, catalog No. 81156) containing 4‐well culture inserts (ibidi GmbH, Germany, catalog No. 80466). After 24 h, the inserts were carefully removed using sterilized tweezers to minimize mechanical injury and avoid abrupt changes in the local environment, as wound healing is known to induce profound metabolic reprogramming. Culture media were then refreshed with RPMI 1640 supplemented with 2% FBS and 1% penicillin‐streptomycin to minimize cell proliferation during migration. Time‐lapse phase‐contrast images were acquired using an EVOS M7000 auto imaging system equipped with a 4× objective (Invitrogen, Thermo Fisher Scientific, the Netherlands) at 20 min intervals over 24 h. The migration ratio was quantified at each time‐point by calculating the cell‐free gap area using automated image segmentation and normalizing it to the initial gap area measured at 0 h.

### 2.4 Live Single‐Cell Sampling

Single cells were generally sampled into 10 µm diameter glass capillaries (Humanix, Japan, CT‐10 µm) using a micromanipulator (Narishige MHW‐103 on MMN‐1 [29]) mounted on a Nikon Ti inverted microscope equipped with an incubator set to 37°C, with 5% CO_2_. The sampling was performed under a 10× objective, with live single cells aspirated into the capillary by applying gentle negative pressure through a manual syringe. The micromanipulator allowed precise, user‐guided movement in three dimensions with fine motor control.

To validate the sampling method, cells from three TNBC cell lines (HCC1143, HCC38, and SUM149PT), cultured at approximately 50 000 cells/mL, were randomly selected for method validation. For single‐cell sampling confirmation, cells in one parallel dish were stained with Hoechst 33342 (0.4 µg/mL) (Thermo Fisher Scientific, the Netherlands, catalog No. 62249) for 30 min prior to the sampling procedure, and the capillaries were imaged under an EVOS microscope at 40× magnification to confirm successful single‐cell capture.

Leader and follower cells were identified based on their position when they were being sampled [14]. The classification was validated further using time‐lapse imaging. Leader cells were located at the migrating front, directly exposed to the extracellular environment and connected to neighboring cells. In contrast, follower cells trailing behind the leaders or moving along the same migration tracks were more surrounded by neighboring cells and exposed to the extracellular environment on fewer sides (Figure S1, Figure 6A). To ensure single‐cell specificity, targeted cells were selected only when individual cell boundaries could be clearly resolved by microscopy, and cells that were tightly clustered with neighboring cells were excluded from sampling. To minimize potential contamination from adjacent cells, sampling was performed from the side of the cell facing the cell‐free (media‐exposed) region, where part of the cell membrane was accessible. For single‐cell sampling during collective migration, cells were microsampled at 18 h (HCC1143) and 16 h (HCC38) post‐wounding. The time points were selected based on the same criteria: the wound gap was not fully closed, a clear leader‐follower pattern was present, and leader cells remained without contact with cells migrating from the opposite side of the gap to ensure reliable single‑cell sampling. For leader‐follower comparisons across two cell lines, each leader cell was paired with a corresponding follower cell sampled from the same wound edge and local migration zone. This ensured that each leader‐follower pair experienced comparable microenvironmental conditions.

**FIGURE 1:**
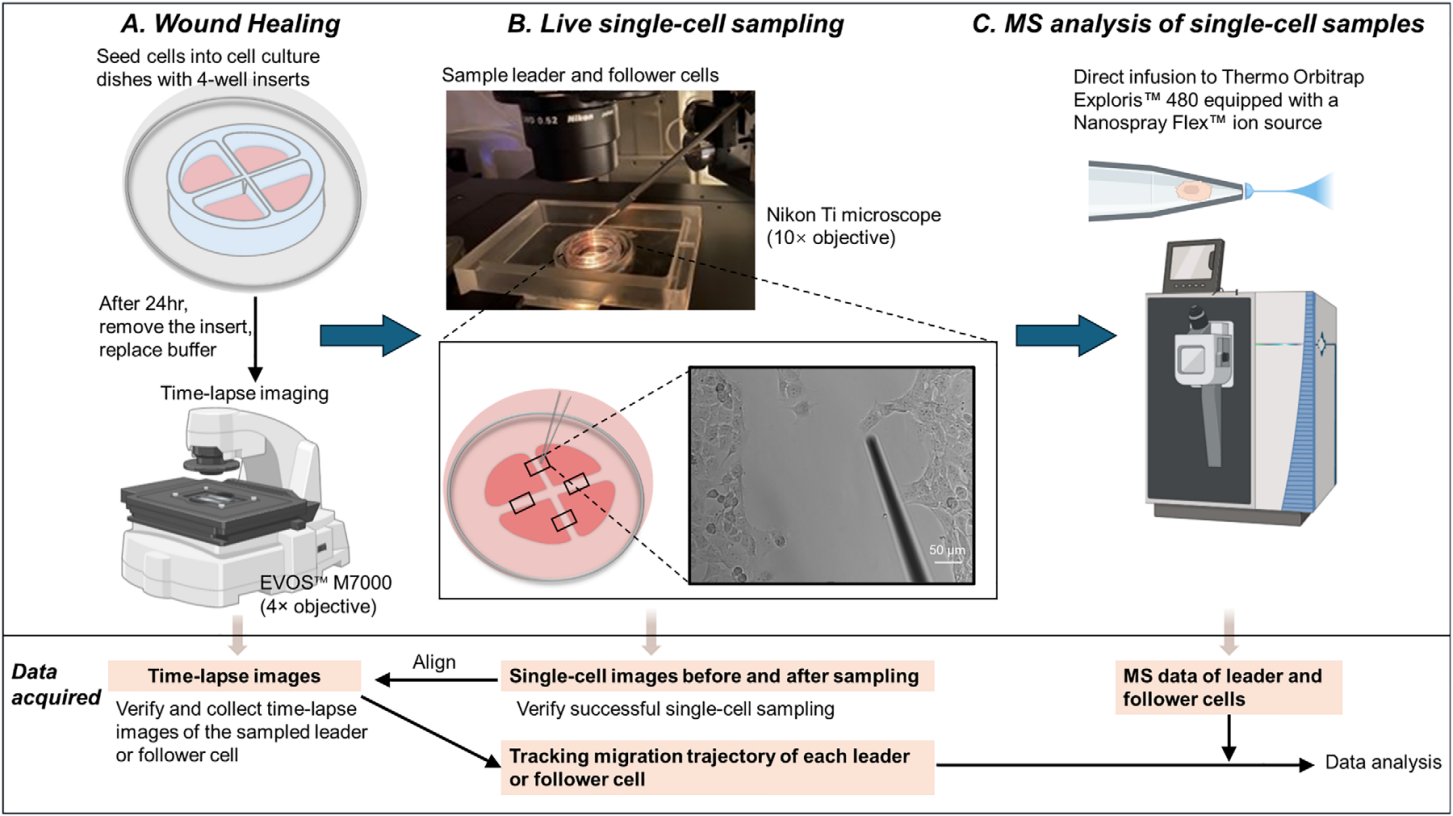
Schematic showing the process of leader and follower cell sampling and MS analysis. After inserts removed, leader and follower single cells were sampled from different zones within the gap area before being analyzed in orbitrap. Scale bar, 50 µm. Since the 4× time‐lapse imaging was conducted on the EVOS system and the subsequent single‐cell sampling was performed on a Nikon Ti microscope, reference markers were drawn on the culture dishes bottom to relocate the same cell on the Nikon Ti and align it to its corresponding position in the EVOS time‐lapse images (Figure S2).

For HCC1143 and HCC38 in collective migration assays, the culture media were replaced with Hank's Balanced Salt Solution (HBSS, Thermo Fisher Scientific, the Netherlands, catalog No. 14175046) supplemented with 5 µM PC(15:0/15:0) immediately before sampling. Leader and follower cells were sampled within 2 h post‐wounding from different zones of the wound area (Figure 1). Phase‐contrast images were taken before and after each single‐cell collection using Nikon Ti microscope with a 10× objective. PC(15:0/15:0) was used as an indicator that the sampling process had occurred. Confirmation of successful single‐cell sampling was achieved through microscopic imaging. After sampling, capillaries containing single cells were fast frozen in liquid nitrogen and stored at −80°C before MS analysis.

### 2.5 Lipid Extraction From Bulk Cells

SUM149PT cells were used to obtain bulk cell extracts for the identification of potential lipid targets. Cells were trypsinized, counted, and then collected by centrifugation at 300 × *g* for 5 min. The cell pellets were washed with ice‐cold PBS and resuspended in 100 µL of water. The cell pellets were subject to two cycles of freeze‐thaw (37°C for 10 min, −150°C for 30 s) to aid cell lysis. The lysis solvent (IPA:ACN:H_2_O = 5:3:2, spiked with 1:480 EquiSPLASH internal standard), containing 0.1% formic acid and 5 mM ammonium formate, was then added to the lysed cells. After incubation on ice for 10 min, the extract was centrifuged at 16,100 × *g* at 4°C for 15 min. The supernatant was collected to get a cell concentration of 4000 cells/µL and serially diluted with the same lysis solvent to approximately 1 cell/µL.

### 2.6 Mass Spectrometry Measurement

The EquiSPLASH internal standard (Table S1) was first used to establish suitable spray voltage and source temperature for ionization. Optimal settings were 1800 V in positive mode and 1600 V in negative mode, with a capillary temperature of 290°C. The optimal collision energy for higher‐energy collisional dissociation (HCD) fragmentation was determined for each lipid class (Table S2).

Bulk cell extracts were analyzed to identify common metabolic features, which were subsequently validated at the single‐cell level. Six concentrations of SUM149PT cell extracts (4000, 500, 125, 31.25, 15.63, and 0.98 cells/µL, prepared by lysis buffer) were analyzed by direct infusion into a Thermo Orbitrap Exploris 480 equipped with a Nanospray Flex ion source (Thermo Fisher Scientific, CA, USA). For each sample, 2.5 µL of cell lysate was loaded into a 10 µm‐diameter nanospray capillary (Cellomics, Japan) from the rear end using a micropipette. MS acquisition was performed in selected ion monitoring (SIM) mode at the resolution of 180 000 full width half maxima (FWHM). The scan range was set to m/z 100–300, 300–600, 600–1000, with peak alignment tolerance of 3 ppm. Features with a correlation coefficient greater than 0.5 between signal intensity and lysate concentration were selected as potential MS^1^ features originating from the cell lysate. Blank removal was performed by applying a median 3‐fold change threshold relative to blanks, followed by a minimum median intensity filter of 2000.

**FIGURE 2:**
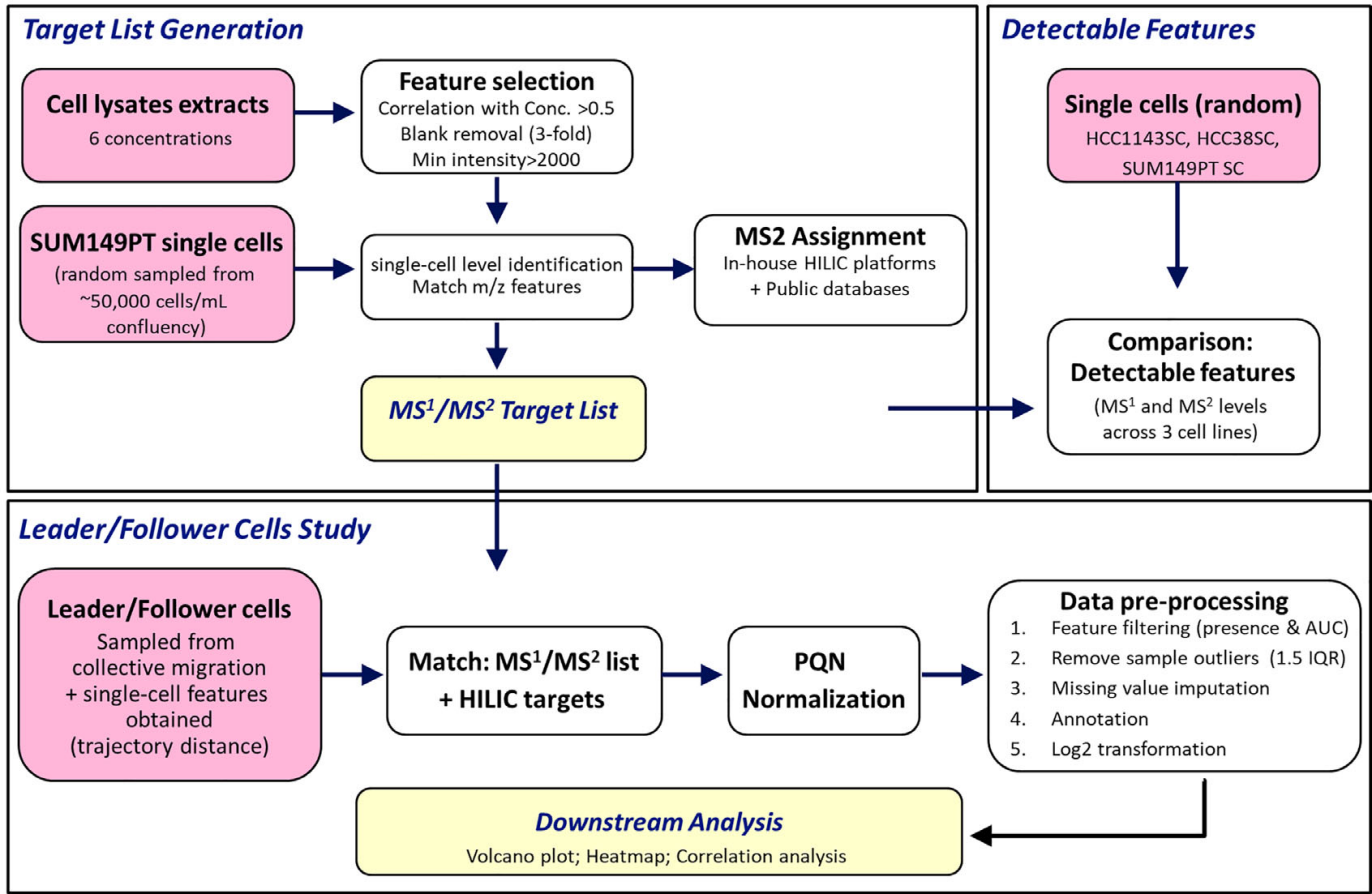
General workflow used in this study.

At the single‐cell level, SUM149PT single cells were analyzed by SIM‐scan MS after backfilling 2.5 µL of lysis solvent into the single‐cell‐containing capillaries. SUM149PT was selected because it exhibits robust lipid signals and has been extensively characterized in our previous study [30], providing a well‐defined metabolic background. HBSS solvent containing PC(15:0/15:0), prepared in the same way, was used as a blank for background removal. The SIM‐scan MS parameters were as follows: positive mode, scan range, m/z 100–300, 300–600, 600–1000; resolution, 180,000; microscans, 1; AGC target, standard (100%, 1e5); maximum injection time, auto; sheath gas flow rate, 0; aux gas flow rate, 0; sweep gas flow rate, 0; spray voltage, 1.8 kV; capillary temperature, 290°C. 1 cell/µL lysate sample was used to optimize AGC target and dynamic exclusion time. Then, a SIM‐ddMS^2^ method was applied to single cells from three cell lines (SUM149PT, HCC1143, HCC38), and further for leader and follower cell analysis. The SIM scan monitored the MS^1^ validated m/z features, and data‐dependent MS^2^ was triggered. All SIM‐ddMS^2^ parameters are summarized in Table S3. The workflow used in this study is illustrated in Figure 2. To minimize potential batch effects, single‐cell measurements were performed using a randomized batch design, with every five biological samples inserted one blank injection. Over five biological replicates were included for each cell line, and detailed metadata are provided in the supplementary single cell metadata file.

### 2.7 Lipid Identification

For MS^1^ features originating from cell lysates, these m/z features were first annotated in the LIPID MAPS structure database [31] to establish a list of candidate lipid species. Then, each precursor ion of the annotated features was assigned a putative fragment ion based on our in‐house HILIC platform targets [32], and by theoretical and experimental fragmentation information from public databases [31, 33]. This process yielded a bulk‐derived MS/MS target list (160 targets in positive mode and 139 targets in negative mode), which formed the basis for subsequent single‐cell lipid analysis.

To expand coverage, this bulk‐derived library was further integrated with an in‐house HILIC MS^1^ reference panel (∼700 lipid features) [32]. Lipid identification was performed using in Skyline‐daily (version 25.1.1.174) [34] using a mass tolerance of ± 5 ppm, following the guidelines of the Lipidomics Standards Initiative (LSI), for which the completed checklist is provided in the Supporting Information. In accordance with the reporting standards for annotations [35], features annotated based on MS^1^ information are level 3 (low confidence), while those supported by MS/MS fragment information are classified as level 2 (medium confidence).

### 2.8 Data Analysis

\Wound‐healing assay images were processed and analyzed in ImageJ (NIH, Bethesda, MD, USA) using a custom macro to enable automated batch analysis. The macro quantified the wound area at each time point, and the exported data were plotted using GraphPad Prism (version 10.1.2). Single‐cell tracking was performed using ImageJ with the manual tracking plugin, and data were further with the ibidi chemotaxis and migration tool 2.0 (ibidi GmbH, Martinsried, Germany) (Supplementary single cell metadata file).

MS data were exported as text files using Xcalibur (version 4.2, Thermo Scientific) and aligned in Markerview (version 1.2.1) with a mass tolerance of 3 ppm. Cells without detectable PC(15:0/15:0) internal standard signals were excluded from further data analysis. Outliers were identified and removed based on the number of detected metabolites per single‐cell sample. Samples with a total number of detected metabolites below the lower bound of the Interquartile Range (IQR) method (Q1‐1.5 × IQR) were discarded, in positive and negative modes separately. Single‐cell data were analyzed using log_2_‐transformed total area under curve (AUC) and normalized using the probabilistic quotient normalization (PQN) method. Blank‐removal was performed by 1.5‐fold threshold and followed by a minimum intensity cutoff of 4000 for single‐cell data analysis. Identified features were further filtered to retain MS^1^ signals present in ≥30% of cells across conditions, and MS^2^ signals >0 presence over 10% for leader and follower cell comparisons.

Principal component analysis (PCA), volcano plots, and heatmaps were generated using R (version 4.3.2). For each cell line, fold changes (FCs) in lipid abundance were calculated using the AUC by dividing the AUC of each lipid in a leader cell by that of its paired follower cell (as defined above). Then FCs from two cell lines were compared to assess differences in leader‐follower contrasts across cell lines using multiple unpaired *t*‐tests in GraphPad Prism (version 10.1.2). The correlation coefficients between single‐cell migration distance and lipid expression levels were calculated using the Spearman method in R (version 4.3.2).

## 3 Results

### 3.1 Development of Acquisition Method for Single Cell Lipid Profiling

**FIGURE 3:**
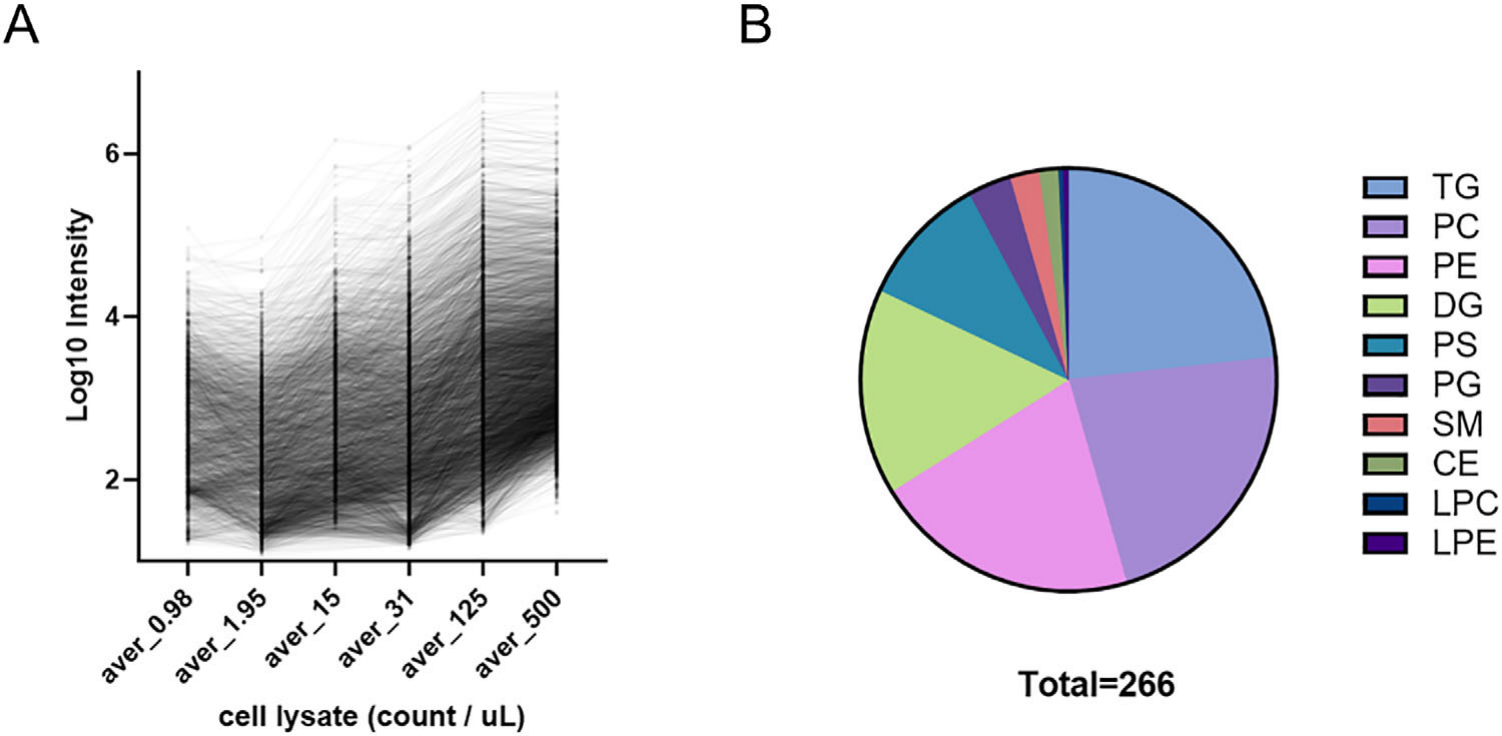
Peak‐list generation using serially diluted cell lysate extracts and SUM149PT single cells. (A) A parallel coordinate plot displays the log_10_‐transformed intensities of common compounds across different cell lysate concentrations (*n* = 5). (B) A total of 266 annotated lipids were identified in single cells (*n* = 24).

Correlation analysis was used to identify MS^1^ features whose signal intensities correlated with lysate concentration (correlation coefficient >0.5; Figure 3A). Applying this filter, together with the blank‐removal criteria described in Section 2.6, yielded 755 m/z features from the bulk SUM149PT cell lysate dataset (Supporting Information S2: Excel.1). To validate which of these 755 potential targets are detectable at single‐cell level, 24 randomly selected SUM149PT single cells (chosen because SUM149PT showed robust lipid signals and was used in our earlier study [30]) were analyzed, and their spectra were matched against the 755‐feature target list. In total, 326 m/z features were detected in the single‐cell data. Of these, 266 were annotated as lipids based on MS^1^ (including TG, PC, PE, and other classes (Figure 3B, Supporting Information S2: Excel. 2)). The full list of MS^2^ features used for annotation and subsequent analysis was compiled in both positive and negative ionization modes with four decimal mass accuracy (Supporting Information S2: Excel.3), providing the curated MS/MS target panel for all subsequent single‐cell lipidomic analysis.

### 3.2 Lipid Profiling Across SUM149PT, HCC1143, and HCC38 Cell Lines

**FIGURE 4:**
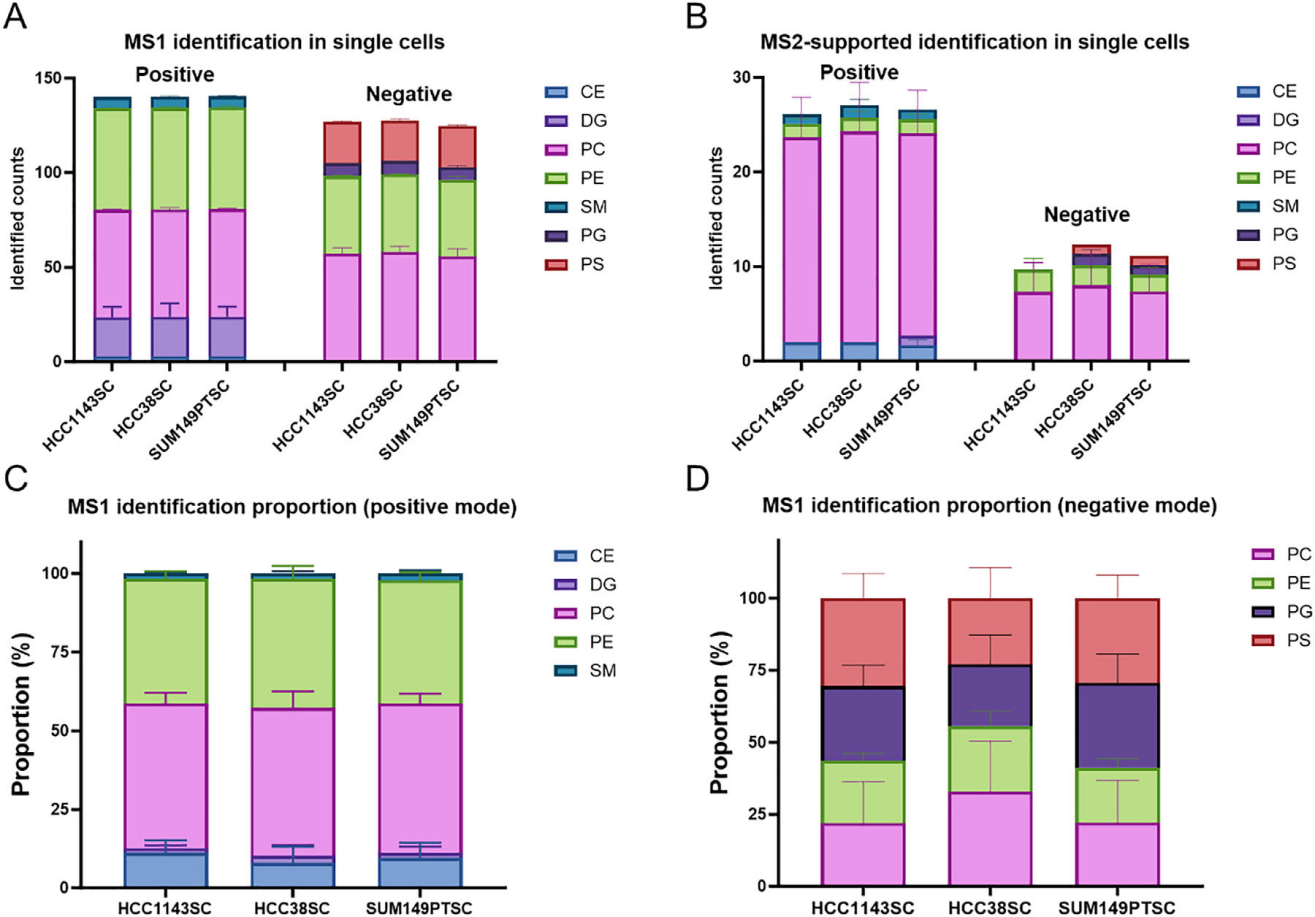
Lipid class distribution across single cells from HCC1143 (*n* = 13), HCC38 (*n* = 21), and SUM149PT (*n* = 12). Identified counts of each lipid class in single cells of three TNBC cell lines in MS^1^ level (A) and targets have fragments (B). (C,D) The distribution of lipids was calculated by total area per class among HCC1143, HCC38, and HCC38 single cells in positive and negative modes.

To study the lipidomic differences in cell lines exhibiting collective migration, we measured the lipid profiles of randomly sampled single cells from SUM149PT, HCC1143, and HCC38 cells. Spectra were processed and matched against the target list in Skyline‐daily software. The MS^1^ data showed signals of cell‐specific lipids in single cells, such as PC(34:1) (∼0.5 *ppm*), PC(38:4) (∼1.8 *ppm*), and PC(36:2) (∼0.7 *ppm*) (Figure S3). In total, 119 targets in positive mode and 125 targets in negative mode were detected in MS^1^ level (Tables S4,S5). At the MS^2^ level, 70 targets in positive mode and 47 targets in negative mode were matched with our curated MS/MS list across the three cell lines (Supporting Information S2: Excel. 4). Both MS^1^‐ and MS^2^‐based identifications revealed that most detected targets belonged to the PC and PE lipid classes (Figure 4A,B).

The distribution of lipid classes was similar across the three cell lines, with PC being the most abundant class, followed by PE (Figure 4C). In negative mode, PE contributed a higher proportion (Figure 4D). Despite individual lipid variation across cell lines, PCA analysis did not reveal clear clustering after applying a 30% presence filter (Figure S4).

### 3.3 Cell Model Selection for Collective Migration

**FIGURE 5:**
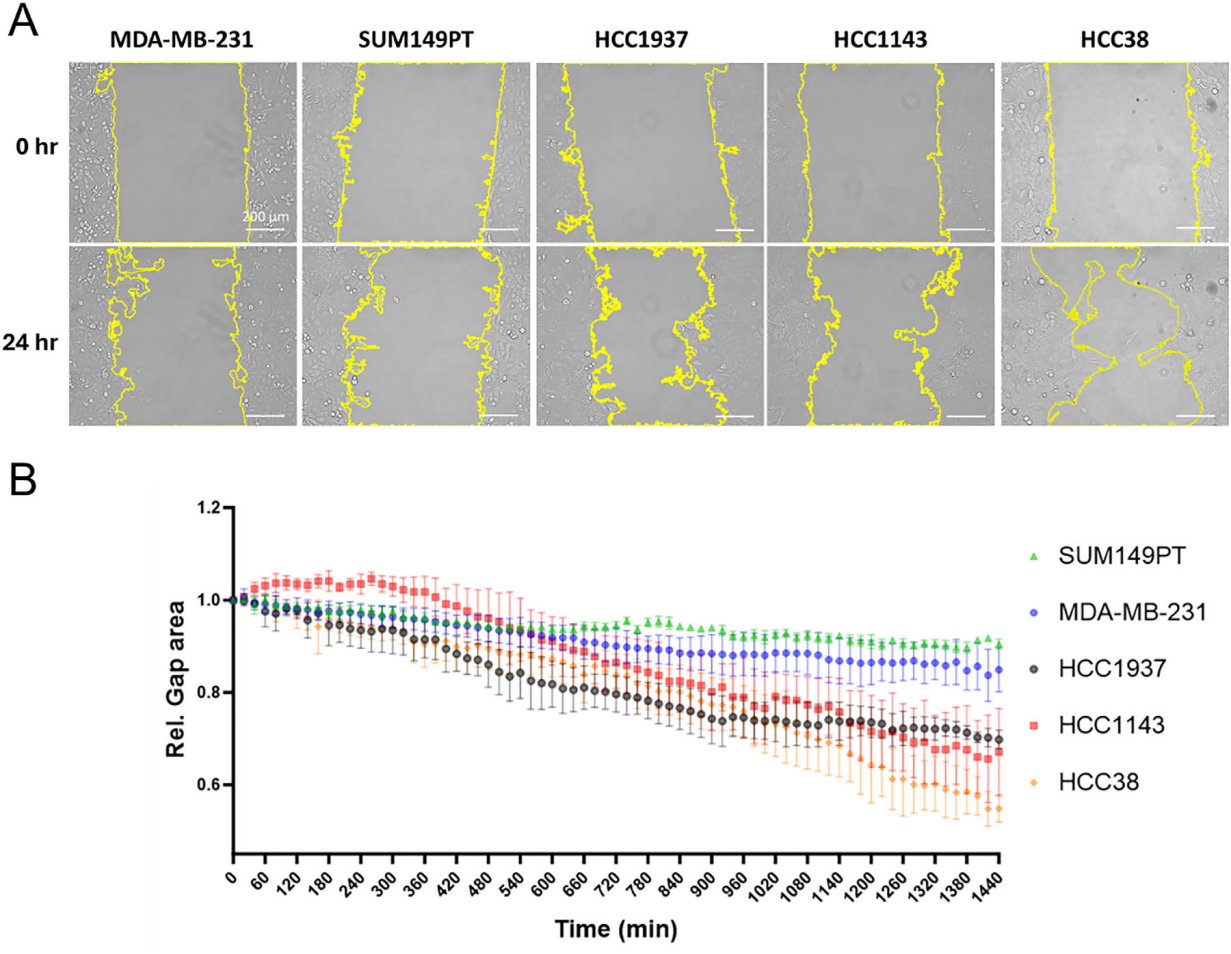
Wound closure dynamic of different cell lines. (A) Wound‐healing representative images at 0 h and 24 h from in vitro wound‐healing assays. Scale bar, 200 µm. (B) Summary graph illustrating relative wound surface area at indicated time points during the wound‐healing assay (*n* = 3).

To identify cell lines that display collective migration behavior, two basal‐A cell lines (HCC1143, HCC1937) and three basal‐B cell lines (SUM149PT, HCC38, and MDA‐MB‐231) were selected to perform the wound‐healing assay. Microscopic observation revealed that four cell lines (HCC1143, HCC1937, SUM149PT, and HCC38) migrated collectively, exhibiting clear leader‐follower organization and maintaining physical connections and cell‐cell interactions (Figure 5A and Figure S5), consistent with the hallmark features of collective cell migration [5]. Among these, HCC1143, HCC1937, and SUM149PT cells showed strong membrane localization of EpCAM, a marker of epithelial integrity and cell‐cell adhesion, whereas HCC38 displayed weak EpCAM expression (Figure S5), likely reflecting its more mesenchymal phenotype (or hybrid EMT [36]). In contrast, MDA‐MB‐231 exhibited weak cell adhesion and predominantly single‐cell migration, in agreement with previous reports [37]. Quantitative analysis of the wound‐healing assay showed that HCC1143 and HCC38 displayed the fastest wound area closure among the tested lines (Figure 5B). Based on these results, HCC1143 and HCC38 were selected for subsequent single‐cell metabolomics analysis.

### 3.4 Lipidomic Profiling Across Leader and Follower Cells

**FIGURE 6:**
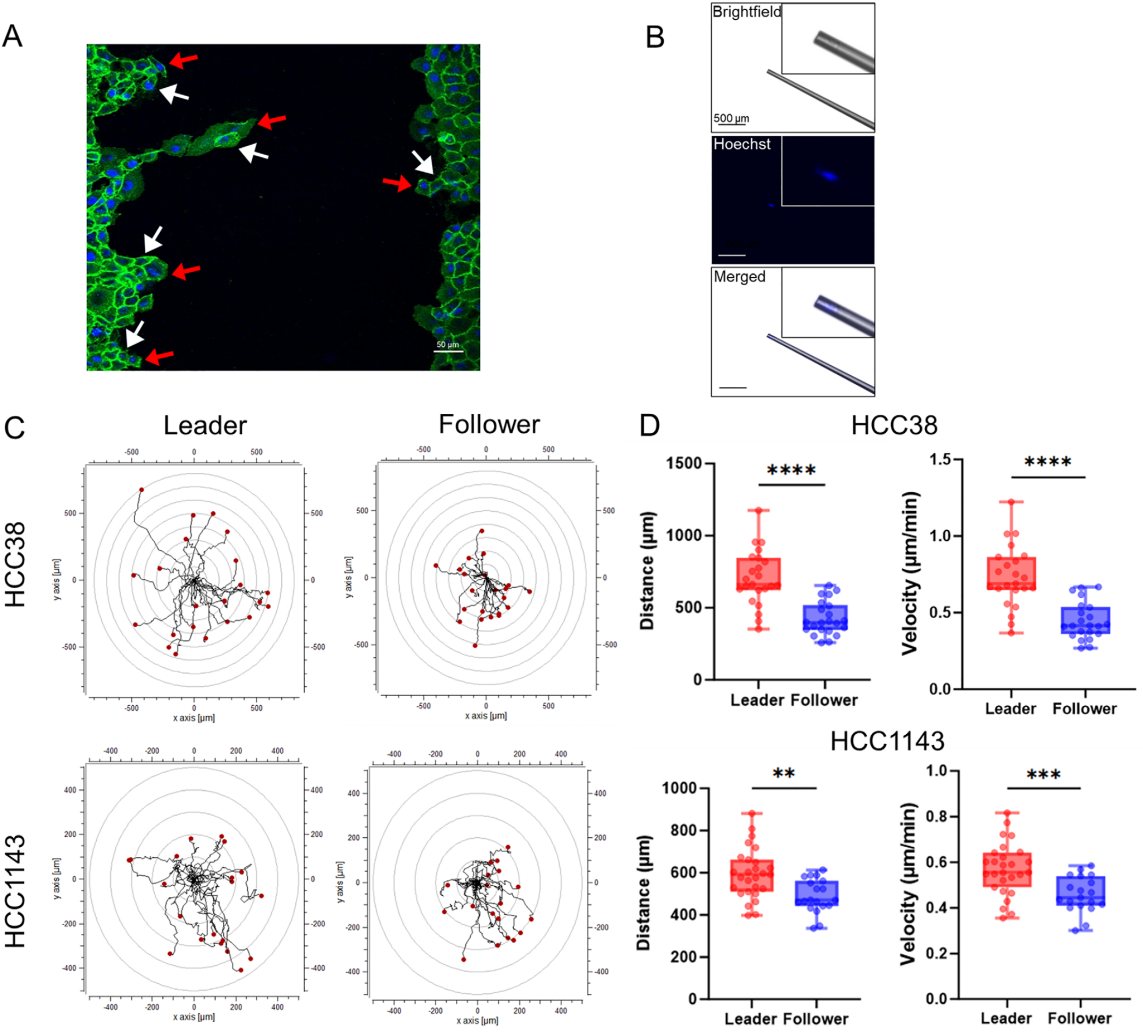
Single‐cell sampling and tracking data of leader and follower cells. (A) Schematic diagram of leader and follower sampling criteria. Cells were stained with Alexa Fluor 488 Anti‐EpCAM antibody (cell membrane, green) and Hoechst 33342 (nuclei, blue). Arrows in red, leader cells. Arrows in white, follower cells. scale bar, 50 µm. (B) Confirmation of a single cell in a capillary. Scale bar, 500 µm. (C) Trajectories of sampled leader and follower cells in HCC38 (top) and HCC1143 (bottom) cell lines. (D) Total distance and mean velocity of all leader (red) and follower (blue) cells. In HCC38, 23 leader cells and 22 follower cells were successfully tracked. In HCC1143, 26 leader cells and 20 follower cells were tracked. ****p < 0.0001, ***p < 0.001, **p < 0.01, using a Student's *t*‐test. Data are presented as min to max all points in GraphPad Prism (version 10.1.2).

Based on the wound healing assays, HCC1143 and HCC38 were selected for single‐cell lipidomic comparison of leader and follower cells. Lipidomic profiling of leader and follower cells was performed according to the criteria mentioned in 2.4 (Figure 1). Typically, a single leader cell at the migration front of the wound was selected first, followed by sampling of a nearby follower cell using the Nikon Ti microscope. In total, 30 leader and 23 follower cells were collected from HCC1143, and 26 leader and 22 follower cells from HCC38. The sampling process is illustrated in Figure 6A,B. Each sampled cell was retrospectively tracked in the time‐lapse images acquired on the EVOS microscope, and the trajectories of the sampled cells were plotted for both cell lines. Leader cells consistently traveled longer distances at higher velocity compared to follower cells (*p* < 0.001, Figure 6C), with this trend being more pronounced in HCC38 than in HCC1143 (Figure 6D).

**FIGURE 7:**
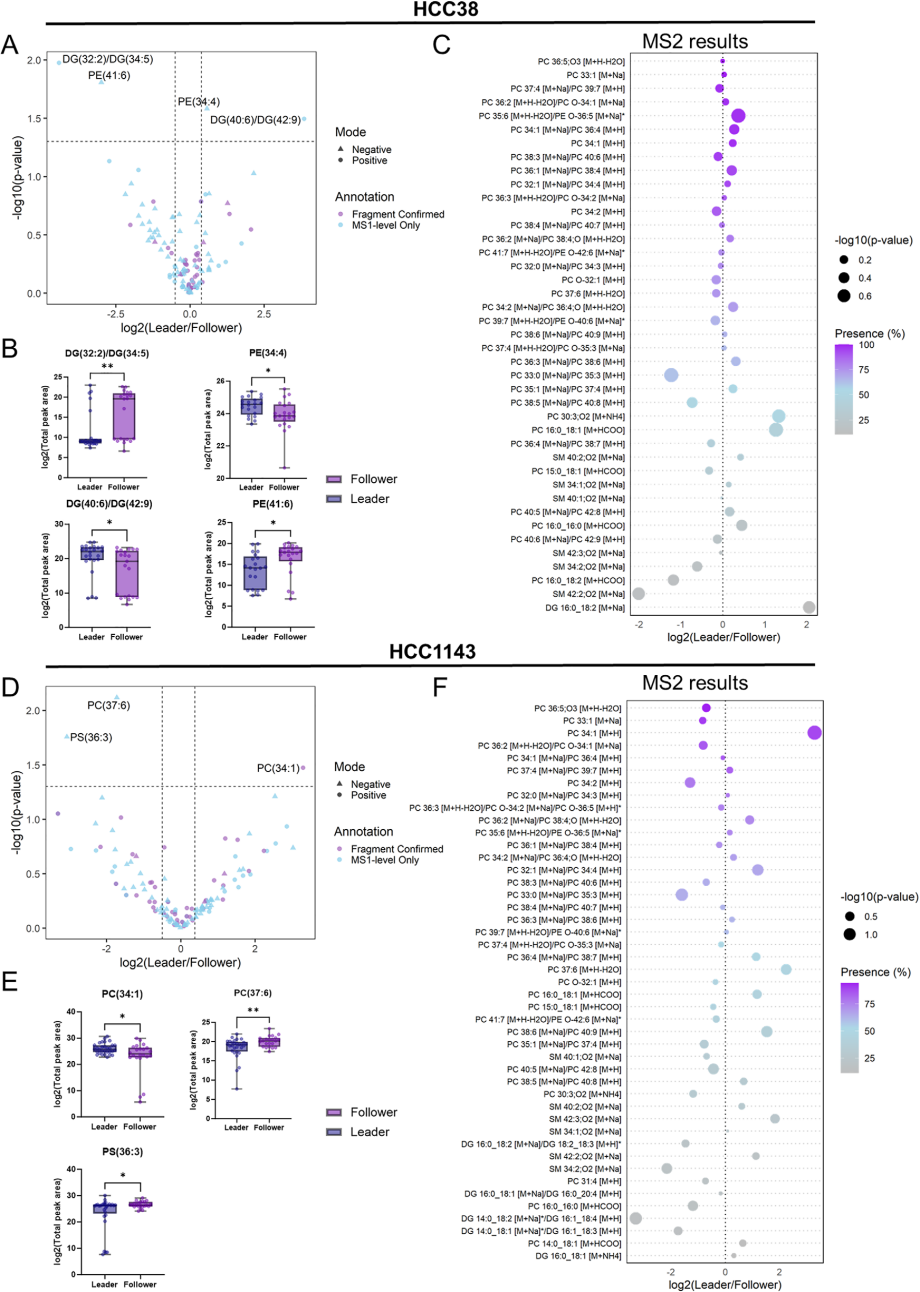
Integration of MS^1^ and MS^2^ data for HCC38 and HCC1143 analysis. (A) Volcano plots of leader and follower single cells from HCC38 cell line. (B) Boxplots of significant targets identified in (A). (C) Targets detectable at the MS^2^ level in HCC38. (D) Volcano plots of leader and follower single cells from HCC1143 cell line. (E) Boxplots of significant targets identified in (D). (F) Targets detectable at the MS^2^ level in HCC1143. In (C) and (F), since a single m/z value can correspond to multiple targets within 5 ppm, those without MS^2^ confirmation are marked with an asterisk (*). Different detection mode and annotation level are annotated. “MS^1^‐level”, features were annotated solely against the predefined target list; “Fragment Confirmed”, lipid species showing matched MS^2^ fragments (>10% presence). Text names were assigned to data points with a fold change threshold 1.3 or 0.7 and *p* < 0.05. Data were log_2_‐transformed and Welch's *t*‐test was applied between leader cells and follower cells groups. **p < 0.01, *p < 0.05, using a Student's *t*‐test.

**FIGURE 8:**
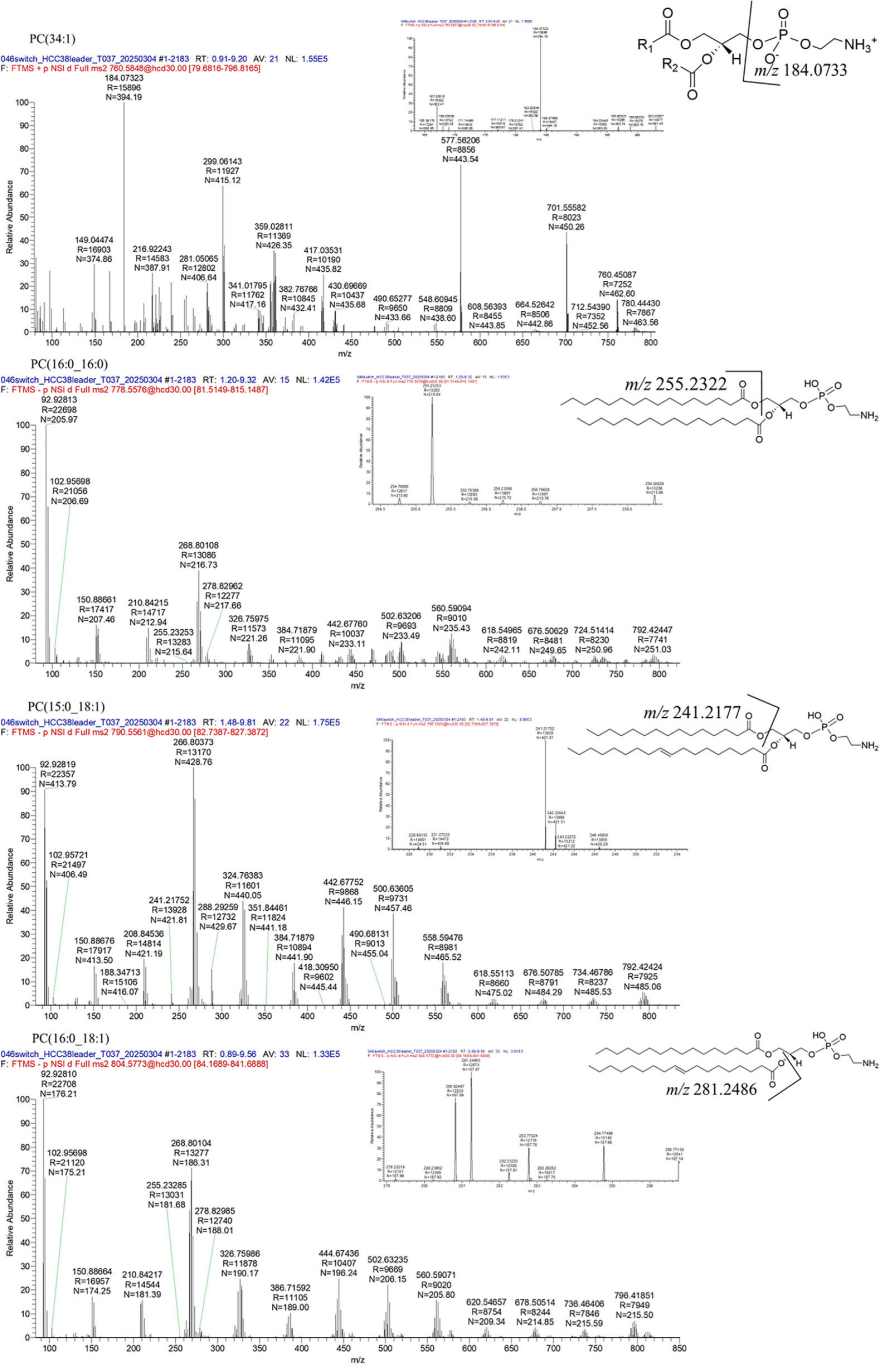
Representative single‐cell MS/MS spectra to show fragments of PC species, either headgroup in positive mode, or fatty acyl chains in negative mode. PC(34:1) is shown as an example, displaying the fragment ion at m/z 184.0733 (precursor m/z 760.5848, −0.39 ppm) in positive ion mode. In negative ion mode, fragment ions at m/z 255.2325 (precursor m/z 778.5576, −2.18 ppm) and 241.2177 (precursor m/z 790.5561, −4.05 ppm) correspond to fatty acyl fragments from PC(16:0_16:0) and PC(15:0_18:1), respectively, and two fragment ions (m/z 255.2329 and 281.2486) were observed for PC(16:0_18:1) (precursor m/z 804.5773, 2.98 ppm).

Following MS measurements and matching against the curated MS^1^/MS^2^ target list, 125 compounds were detected at the MS^1^ level in HCC38 (level 3). Four compounds showed significant differences (*p* < 0.05) in HCC38 between leaders and followers: DG(32:2)/DG(34:5), PE(41:6), PE(34:4), and DG(40:6)/DG(42:9) (level 3) (Figure 7A). Boxplot analysis revealed up‐ or down‐regulation of these compounds in leader versus follower (Figure 7B). Fold changes and p‐values of significant features (*p* < 0.05) were included in Table S6. After applying a fragment‐level filter, 41 compounds were retained (Figure 7C). In positive mode, 30 PC and 6 SM species were detected (level 2), with 184.0733 ion fragments indicative of the phosphocholine headgroup. Side‐chain fragments for PC(15:0_18:1), PC(16:0_16:0), PC(16:0_18:1), and PC(16:0_18:2) were detected in negative mode (Figure 8 and Figure S6) (level 2).

For HCC1143, three compounds‐PC(34:1), PC(37:6), and PS(36:3)‐displayed significant differences (*p* < 0.05) between leaders and followers among 130 compounds after data filtration, with PC(34:1) showing a matched MS^2^ matched fragment (Figure 7D,E). A total of 44 MS^2^ spectra were obtained in HCC1143 with over 10% presence across the detected samples, dominated by PC species (Figure 7F) (level 2). In positive mode, 29 PC, 6 SM, and 5 DG species were detected, with PC(14:0_18:1), PC(16:0_16:0), PC(15:0_18:1), and PC(16:0_18:1) showing fatty acyl fragments in negative mode (Figure 8 and Figure S6) (level 2).

### 3.5 MS^1^ Putative Identification Profiling for Leader and Followers (Level 3)

**FIGURE 9:**
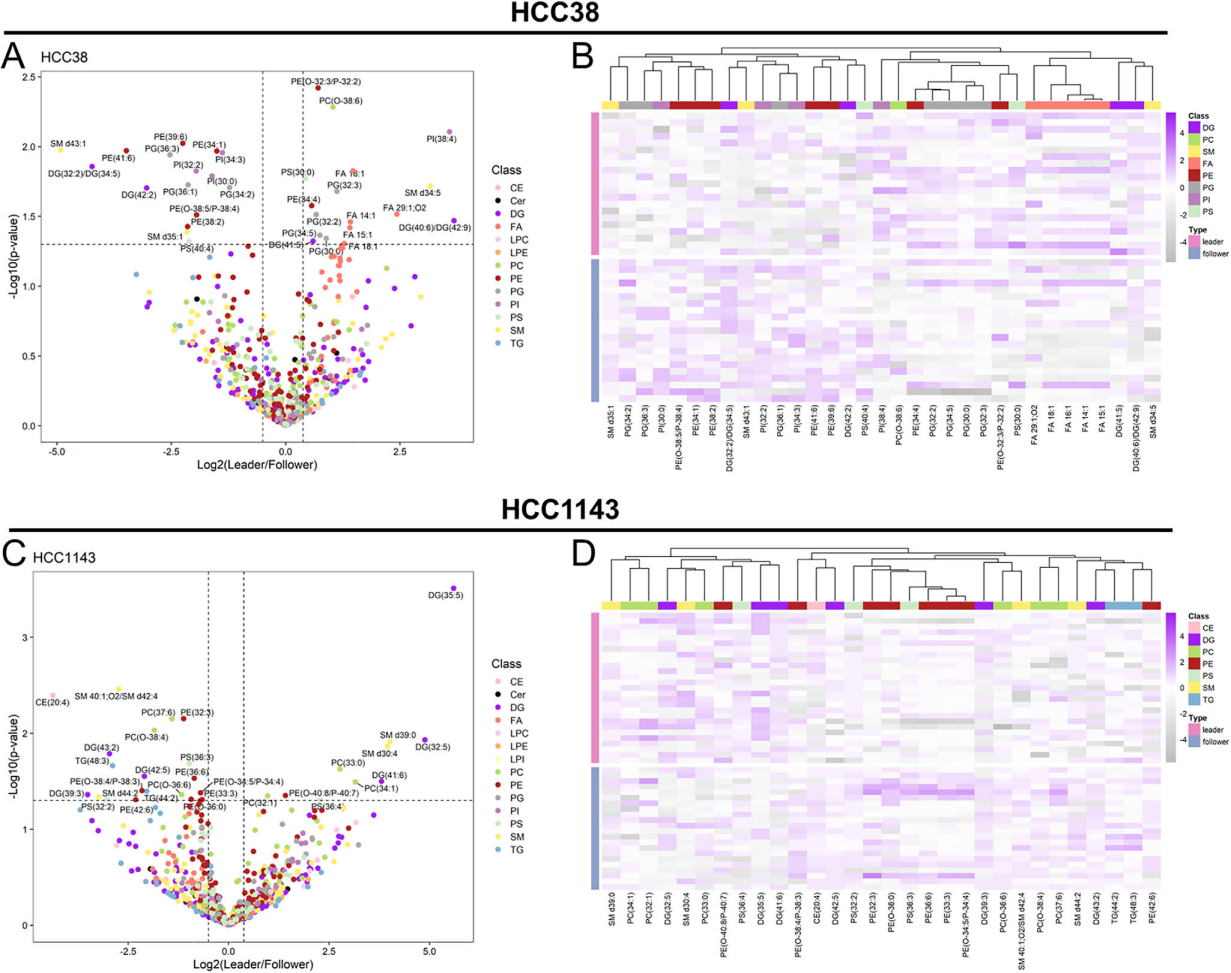
Volcano plots and heatmaps showing alterations between leader and follower cells. (A) Volcano plot showing changes of lipid species in leader and follower cells in HCC38 cell line. (B) Heatmap of significantly changed metabolites in (A). (C) Volcano plot showing changes of lipid species in leader and follower cells in HCC1143 cell line. (D) Heatmap of significantly changed metabolites in (C). Data were log_2_‐transformed, and Student's *t*‐test was applied between leader cells and follower cells groups. MS^1^ annotated names were assigned to data points with fold change threshold 1.3 or 0.7 and *p* < 0.05.

To expand lipidomic coverage, MS^1^ data from single cells were matched against (i) the bulk‐derived MS^1^ target list generated from cell lysates and (ii) an in‐house reference panel containing lipid species annotated at the sum composition level [32] (total number of carbons and double bonds across all fatty acyl chains) (level 3) (Supporting Information S2: Excel.5). For all matched features, volcano plots were generated per cell line to compare leader and follower populations. In HCC38, significant alterations (*p* < 0.05) were observed across multiple lipid classes: several DG, PE, PI and PG species showed lower abundance in leader cells, whereas fatty acids and subsets of PG and DG species were increased in leaders (Figure 9A) (Supporting Information S2: Excel. 6). In HCC1143, fewer species were differentially abundant than in HCC38, with significant changes (*p* < 0.05) mainly within PE, PC and DG classes; several DG and PE species were reduced in leaders, while a subset of PC and DG species was elevated (Figure 9C). Heatmaps highlighted substantial single‐cell heterogeneity in lipid profiles within both leader and follower populations in each cell line (Figure 9B,D). However, when averaged per group, no significant differences were detected in the mean abundance of these species or in lipid class ratios (PC/PE, PC/LPC, PE/LPE, DG/PC, DG/PE, and DG/TG) in either cell line (Figure S7). After integrating the significant compounds (*p* < 0.05) between leader and follower cells from both lysate‐derived targets and the in‐house target list above, correlation coefficients were calculated and ranked to investigate potential relationships between lipid alterations and single‐cell migration ability. The correlation analysis between individual lipid species and the total migration distance of each single cell revealed that most compounds showed low correlation coefficients. A few lipids exhibited relatively higher correlations with migration distance in HCC38 cells, and annotated lipids showing a correlation coefficient greater than 0.3 or less than −0.3 and *p* < 0.05 in correlation analysis were shown in scatter plots (Figure S8).

To directly compare leader‐follower contrasts between cell lines, each leader cell was paired with a follower cell sampled from the same wound edge and local migration zone, and fold changes in lipid abundance were calculated for each leader‐follower pair. Comparing these leader‐to‐follower fold changes revealed 17 targets with significantly different leader‐to‐follower contrasts between the two cell lines (*p* < 0.05). Among these, 7 PE species and 3 PG showed higher fold changes in HCC1143 pairs compared to HCC38, indicating cell‐line‐specific metabolic specialization (Figure S9).

## 4 Discussion

In this study, we systematically investigated the lipidomic profiles associated with leader‐follower organization during collective migration in two TNBC cells using a label‐free, single‐cell mass spectrometry method. By integrating wound‐healing assays, time‐lapse imaging, and single‐cell mass spectrometry, we demonstrated that leader and follower cells have cell line‐specific lipidomic differences across multiple classes. Our manual live single‐cell sampling strategy, performed at wound edges under microscopic guidance, enabled lipidomic analysis while preserving migratory phenotypes.

Single leader and follower cells were sampled from two highly migratory basal‐like breast cancer lines, HCC1143 and HCC38. Each sampled leader or follower cell was retrospectively tracked, and trajectory analysis showed that the sampled leader cells migrated farther and faster than follower cells, confirming phenotypic differences (Figure 6). However, in our data, leader‐follower lipidomic differences were cell line‐specific: HCC38 leaders exhibited significant alterations in 2 PE and 2 DG species (*p* < 0.05), whereas HCC1143 leaders displayed distinct changes in 2 PC and 1 PS species (Figure 7) (level 3). These results indicate that lipid remodeling during collective migration might be cell‐line dependent, reflecting the metabolic diversity among basal‐like breast cancer subtype.

Across both cell lines, significant alterations in the abundance of multiple lipid classes were revealed in MS^1^‐based putative identification (*p* < 0.05). In HCC38, the most significantly altered lipids were PEs and fatty acids, with most PEs showing decreased levels and fatty acids showing increased levels in leader cells. Besides, several PG and PI species were reduced in HCC38 leaders (level 3). The reduction in structural lipids (PE, PG, some DG) together with higher fatty acids suggests that enhanced lipolysis and fatty acids could be used for ATP production (via β‐oxidation) and signaling [38]. It also suggests that leader cells of HCC38 might be actively turning over membranes and using lipids as a flexible energy reservoir during migration, a pattern reported in aggressive cancer cells with high lipid metabolic flux rather than simple accumulation [39]. In HCC1143, fewer significantly altered targets were observed (*p* < 0.05), but a similar direction appears: PE species showed lower levels in leader cells while a subset of PC and DG species is higher (Figure 9) (level 3), indicating that membrane composition is being reshaped rather than globally increased or decreased. To further explore the relationship between lipid profiling and migratory behavior, we performed correlation analysis between single‐cell lipid levels and migration distance. The overall correlation coefficients were low, and patterns differed between HCC38 and HCC1143 (Figure S8), suggesting that the standard thresholds for correlation strength may need adjustment for single‐cell data, where biological variability is inherently high.

Overall, PC and PE were the most abundant species detected across all measured cell lines (Figure 4). PC and PE play crucial roles in defining membrane curvature, fluidity, and cellular dynamics [40, 41]. PC is the most abundant membrane glycerophospholipid and has been implicated in enhanced motility and invasion in various cancers. On the other hand, PE contributes to membrane fusion, cell viability, and autophagy [42]. PE lipids also affect membrane curvature important for vesicle formation, influencing intracellular trafficking and dynamic membrane remodeling during migration [41]. Furthermore, the PC/PE ratio, often considered a determinant of membrane asymmetry and cytoskeletal reorganization [42, 43], showed no consistent trend between leader and follower cells. Studies indicated that DG acts as a pivotal lipid signaling molecule influencing membrane dynamics and cell motility [44]. DG/PC and DG/PE ratios were higher in leader cells in both cell lines; however, the differences were not statistically significant (Figure S7). Given that DG serves as a key lipid signaling molecule activating protein kinase C (PKC) and modulating cytoskeletal rearrangement [40], changes in the DG/PC and DG/PE ratios could modulate PKC activation and downstream signaling cascades that regulate cytoskeletal remodeling and cancer cell migration [40].

For the MS^2^‐confirmed compounds, only PC(34:1) showed significant leader‐follower differences in HCC1143 (*p* < 0.05) (level 2). Since measurements were done in both positive and negative mode, side‐chain level identification based on MS^2^ fragmentation is possible. The species annotated as PC(34:1) corresponded to PC(16:0_18:1), while PC(33:1) was assigned as PC(15:0_18:1) in both cell lines (level 2). Similarly, the annotation of PC(32:0) was consistent with PC(16:0_16:0) (Figure 7 and Figure S6) (level 2). This is consistent with previous single‐cell study of breast cancer cell MCF‐7 [28]. These results showed that it is possible to perform more comprehensive structure elucidation of lipids on the single‐cell level for better biomarker identification.

Our study has several limitations. Manual phenotypic identification and single‐cell sampling constrained throughput and introduced operator variability. In addition, not all targets could be measured using MS/MS due to the low signal, a limitation that has also been reported in previous studies using direct capillary sampling of single cells as well as live‐cell imaging approaches [24, 25]. Furthermore, lipid annotations in this study are primarily based on MS^1^‐level information; therefore, future validation using MS/MS or complementary approaches will be necessary to improve identification confidence. This means that not all of the metabolic divergence between leader and follower cells sampled from the same environment could be identified with high confidence. Nonetheless, our results support the notion that collective migration is a shared phenotype underpinned by heterogeneous metabolic programs, as mentioned in previous work [45], while providing new single‐cell‐level insights into lipidomic differences between leader and follower cells that may contribute to metastatic behavior. Despite these limitations, SIM‐ddMS^2^ acquisition mode generated 120 compounds in one single cell in MS^1^ and about 70 compounds with a matched fragment in MS^2^, suggesting that single‐cell MS/MS lipid identification is achievable.

On the other hand, it is also important to note that leader and follower identities are transient cellular states rather than fixed phenotypes [46]. Leaders and followers can interconvert and change roles, often through a dynamic, “relay‐like” swapping process that sustains forward invasion through complex extracellular matrix [47, 48]. This leader‐follower switching is regulated by multiple factors, including cellular energetic status and cell cycle phase during breast cancer invasion. For instance, once a leader cell's energy levels are depleted, a follower cell may assume the leader position, maintaining continuous invasive capacity [48]. Therefore, single cells sampled from each phenotype in this study likely represent dynamic snapshots of collective behavior rather than stable subpopulations. This variability underscores the complexity of functional states in collective migration, and highlights the challenge of definitively attributing metabolic features to specific phenotypes. Without explicit cell isolation or spatial resolution, bulk‐level interpretations in heterogeneous systems may obscure subtype‐specific biological signals. This is particularly relevant in dynamically interconverting cellular states, where population‐level measurements can mask phenotype‐specific metabolic heterogeneity and lead to signal averaging effects. Future improvements in automated single‐cell isolation, high‐sensitivity MS acquisition, and spatial metabolomics integration will be critical to enhance throughput, reproducibility and identification confidence. Besides, combining single‐cell lipid profiles with immunostaining of epithelial markers [14] or cell cycle reporter [49], may provide a valuable strategy to distinguish leader‐follower heterogeneity across invasion fronts. Such multimodal approaches will enable researchers to capture the temporal evolution of leader‐follower state transitions and more precisely link metabolic rewiring to dynamic migratory behaviors.

## 5 Conclusion

Single‐cell lipid profiling revealed leader‐follower specific lipid alterations during collective migration, particularly involving PE, PC, and DG classes. These findings suggest that lipid metabolism contributes to collective behavior in breast cancer cells, highlighting cell line‐specific lipidomic changes of leader cells. Technically, this work demonstrates that direct infusion‐based single‐cell lipid profiling is feasible under high‐confluency conditions, providing a route to explore dynamic cellular phenotypes in their native microenvironments.

## Funding

This research was supported by NWO XS grant [number OCENW.XS23.1.070.], China Scholarship Council [No. 202206280025].

## Conflicts of Interest

The authors declare that they have no conflicts of interest.

## Supporting information




**Supporting File 1**: advs75862‐sup‐0001‐SuppMat.pdf.

**Supporting File 2**: advs75862‐sup‐0002‐data.zip.

## Data Availability

Metabolomics data generated in this study have been deposited in EMBL‐EBI MetaboLights with the identifier MTBLS13405 and are accessible at https://www.ebi.ac.uk/metabolights/MTBLS13405. Time‐lapse imaging data and single‐cell capture images for breast cancer cell lines study including the macron are available on Zenodo (10.5281/zenodo.17726828). All additional files referenced in the manuscript are provided in the Supplementary files and Supplementary Word document.
