# Supplementary material for: Imaging‐Guided Live Single‐Cell Lipid Profiling of Leader and Follower Cells During Collective Migration of Triple‐Negative Breast Cancer Cells
Source: Adv Sci (Weinh). 2026 Jun 16:e75862. Online ahead of print. doi: 10.1002/advs.75862 (PMC13336123; doi:10.1002/advs.75862)
Supplement: Supplementary file 1 — Supporting File 1: advs75862‐sup‐0001‐SuppMat.pdf. [file ADVS-9999-e75862-s001.pdf]

Supplementary material for:

Imaging-guided live single-cell lipid profiling of leader and follower cells during collective migration of triple-negative breast cancer cells

Xiaoyue Huang[1], Judit González Riera[1], Mai Nguyen[1], Jeremy Broekhuis[2], Sylvia E. Le Dévédec[3,*], Ahmed Ali[1,*], Thomas Hankemeier[1,*]

⋆ corresponding authors' emails: s.e.ledevedec@lacdr.leidenuniv.nl, ali@lacdr.leidenuniv.nl, Hankemeier@lacdr.leidenuniv.nl

[1]Metabolomics and Analytics Centre, Leiden Academic Centre for Drug Research, Leiden University, 2333 CC Leiden, The Netherlands

[2]Institute of Biology, Leiden University, 2333 CC Leiden, The Netherlands

[3]Division of Cell Systems and Drug Safety, Leiden Academic Centre for Drug Research, Leiden University, 2333 CC Leiden, The Netherlands

Single cell metadata, LSI checklist, and Supplementary Excel 1-6 are in separate files:

Single cell metadata Single-cell migration tracking data and metadata for HCC38 and HCC1143 cells.

LSI checklist Lipidomics Standards Initiative checklist.

Supplementary Excel.1 Potential targeted metabolite list derived from bulk cell lysates. This table contains the lipids identified from bulk cell lysates, serving as the reference library for single-cell annotation. "Correlation coefficient" column provides the Pearson correlation coefficient between the m/z signal intensity and the gradient concentrations of cell lysates.

Supplementary Excel.2 Putative lipid assignments based on MS1 accurate mass. A detailed list of lipid species annotated in cell lysates using MS1 information.

Supplementary Excel.3 The list of MS2 features used for annotation. This table contains the MS2 fragment ions used to elevate metabolite annotations to MSI Level 2 (medium confidence). The table includes precursor ion (m/z), name, molecular formula, charge, ion adduct, fragment ion (m/z).

Supplementary Excel.4 Metabolite data matched against the target list from Excel.3.

Supplementary Excel.5 Mass accuracy (ppm) distribution for detectable features. Related to Figure 9.

Supplementary Excel.6 Statistically significant metabolic features in HCC38 and HCC1143. Related to Figure.9.

Classified as Internal | Intern

Figures and tables included in this Supplementary Material:

Figure S1. Representative images of leader or follower cell before and after sampling.

Figure S2. Time-lapse images acquired with the 4× objective aligned with single-cell images taken before and after sampling using the 10× objective.

Table S1. Information of each internal standard.

Table S2. Optimal HCD of each internal standard at 290 °C.

Table S3. Mass spectrometry parameters used for leader and follower cell analysis in the SIM-MS/DDA method.

Figure S3. Representative MS^1 spectra of a single cell showing the presence of signals for MS^1 annotated lipids PC(34:1), PC(38:4) and PC(36:2) in SUM149PT, HCC1143, and HCC38 cell lines.

Table S4. Total detected counts of lipids across all single cell samples in positive ion mode.

Table S5. Total detected counts of lipids across all single cell samples in negative ion mode.

Figure S4. PCA plots of MS1 targets for HCC1143, HCC38, and SUM149PT single cells in positive and negative ionization modes.

Figure S5. Collective migration behavior and EpCAM expression patterns in five TNBC cell lines.

Table S6. Fold changes and p-values of significant features that were matched to lysates-derived target list in HCC38 and HCC1143.

Figure S6. Representative MS/MS spectra of PC species in single cells from HCC38 and HCC1143. Zoomed-in views.

Figure S7. Volcano plots of average expression levels of each lipid class and class ratio alterations between leader and follower cells in HCC38 (above) and HCC1143 (down).

Figure S8. Barplots of the ranked coefficients and scatter plots of highly correlated targets ($p < 0.05$) between individual lipid compounds and the migration accumulated distances of single cells in HCC38 (A) and in HCC1143 (B).

Figure S9. Heatmap (A) and violin plots (B) showed significant leader-to-follower differences between HCC38 and HCC1143 cell lines ($p < 0.05$).

Classified as Internal | Intern

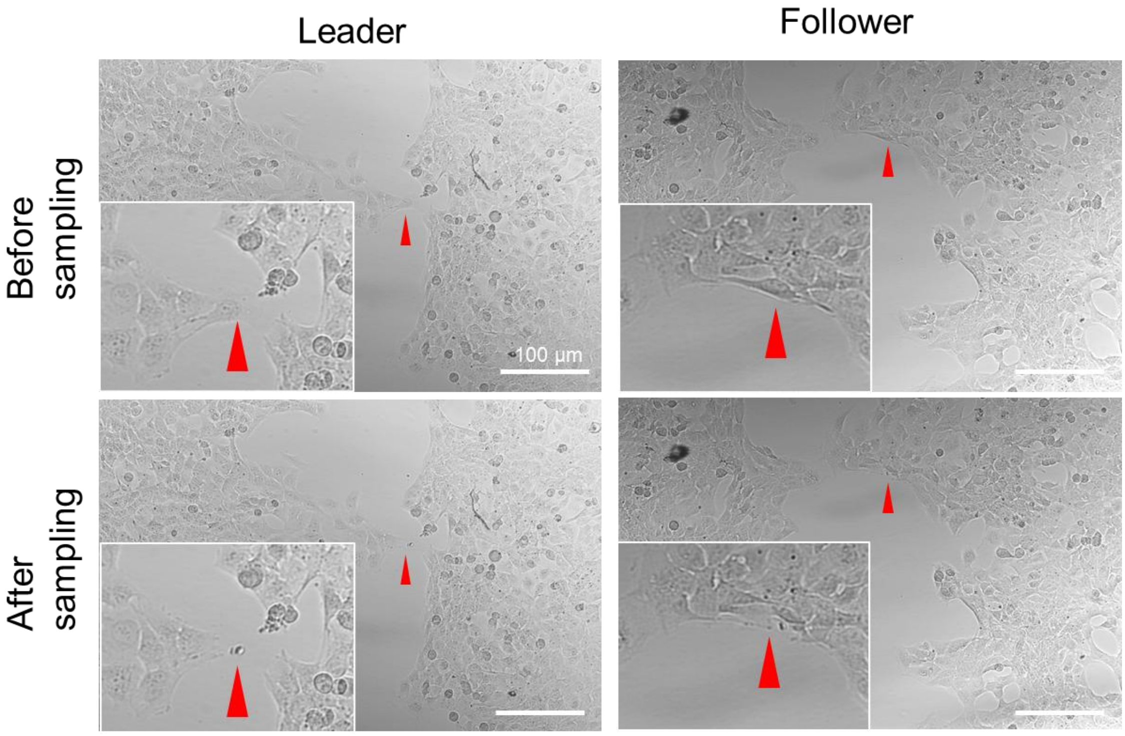

Figure S1. Representative images of leader or follower cell before and after sampling. Arrowhead, the single cells that were sampled. Scale bar, 100 µm.

Classified as Internal | Intern

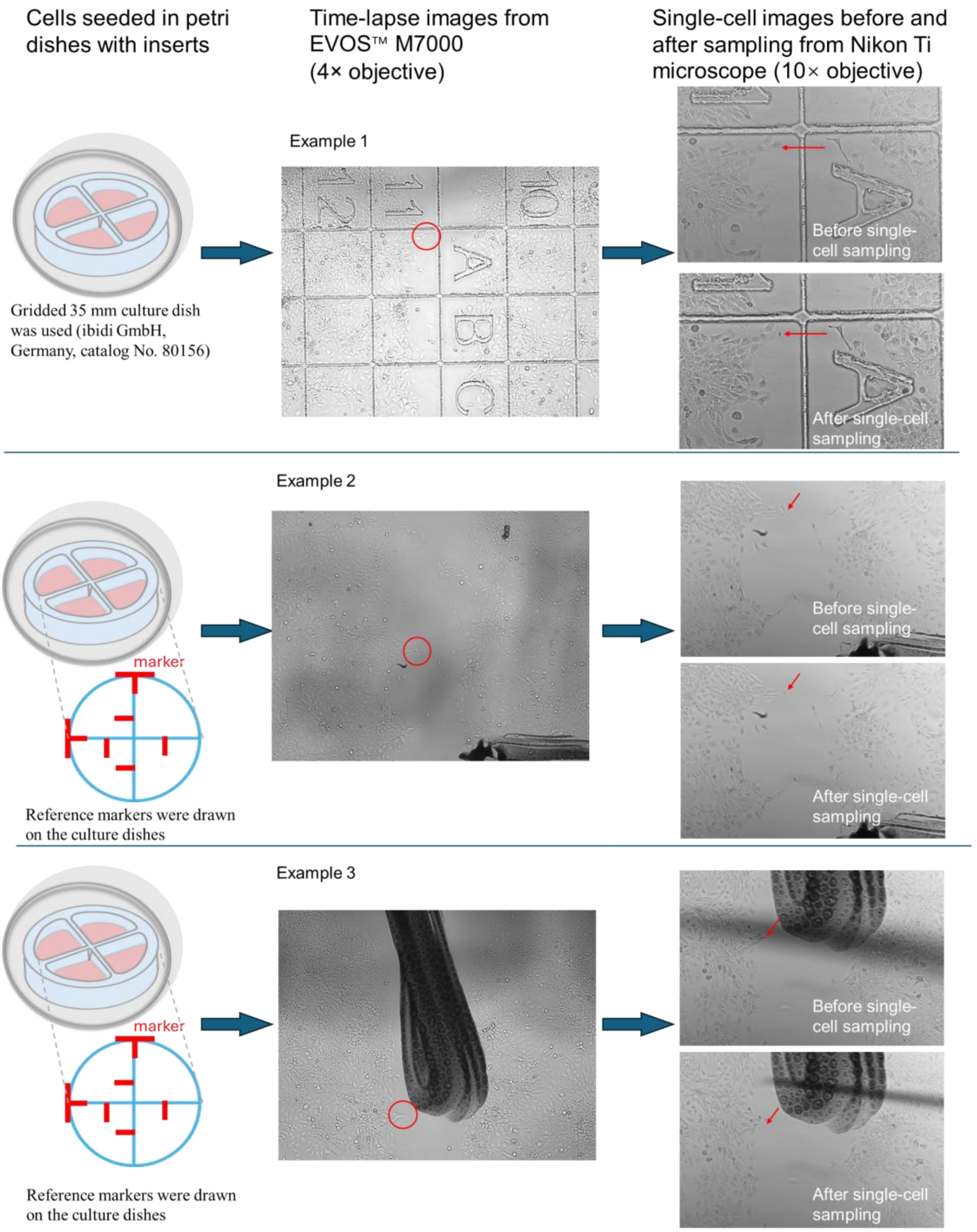

Figure S2. Time-lapse images acquired with the 4× objective aligned with single-cell images taken before and after sampling using the 10× objective.

Table S1. Information of each internal standard.

EquiSPLASH standard	Molecular formula	m/z pos (+H adduct)	m/z pos (+NH4 adduct)
15:0-18:1-d7-PC	$C_{41}H_{73}D_{7}NO_{8}P$	753.6139	
15:0-18:1-d7-PE	$C_{38}H_{67}D_{7}NO_{8}P$	711.5670	
18:1-d9 SM	$C_{41}H_{72}D_{9}N_{2}O_{6}P$	738.6475	
18:1-d7 Lyso PC	$C_{26}H_{45}D_{7}NO_{7}P$	529.3999	
18:1-d7 Lyso PE	$C_{23}H_{39}D_{7}NO_{7}P$	487.3530	
15:0-18:1-d7-15:0 TG	$C_{51}H_{89}D_{7}O_{6}$		829.7990
15:0-18:1-d7 DG	$C_{36}H_{61}D_{7}O_{5}$		605.5850

Table S2. Optimal HCD of each internal standard at 290 °C.

IS	15:0-18:1-d7-PC	15:0-18:1-d7-PE	15:0-18:1-d7-15:0 TG	18:1-d9 SM	18:1-d7 Lyso PC	18:1-d7 Lyso PE	18:1-d7-cholesterol easter	15:0-18:1-d7 DG	15:0-18:1-d7-PG
HCD (%, positive mode)	30	20	20	30	30	20	10	30	
HCD (%, negative mode)	30	30			40	30			50

Classified as Internal | Intern

Table S3. Mass spectrometry parameters used for leader and follower cell analysis in the SIM-MS/DDA method.

Parameters	Values
Spray current	~ 0.2 µA
Temperature	290 °C
Experiment time	10 min
Dynamic exclusion time	(after 3 times within 60 s) 60 s
AGC target	150% , 1.5e5
HCD	Same as Table S2
Loop count (Top N)	10
Isolation window (m/z)	2
SIM resolution	240,000 FWHM
MS2 resolution	15,000 FWHM
Exclude isotopes	on
Easy-IC	on
SIM scan mass error ppm	10
ddMS2 mass error ppm	5
ddMS2 intensity threshold	4000

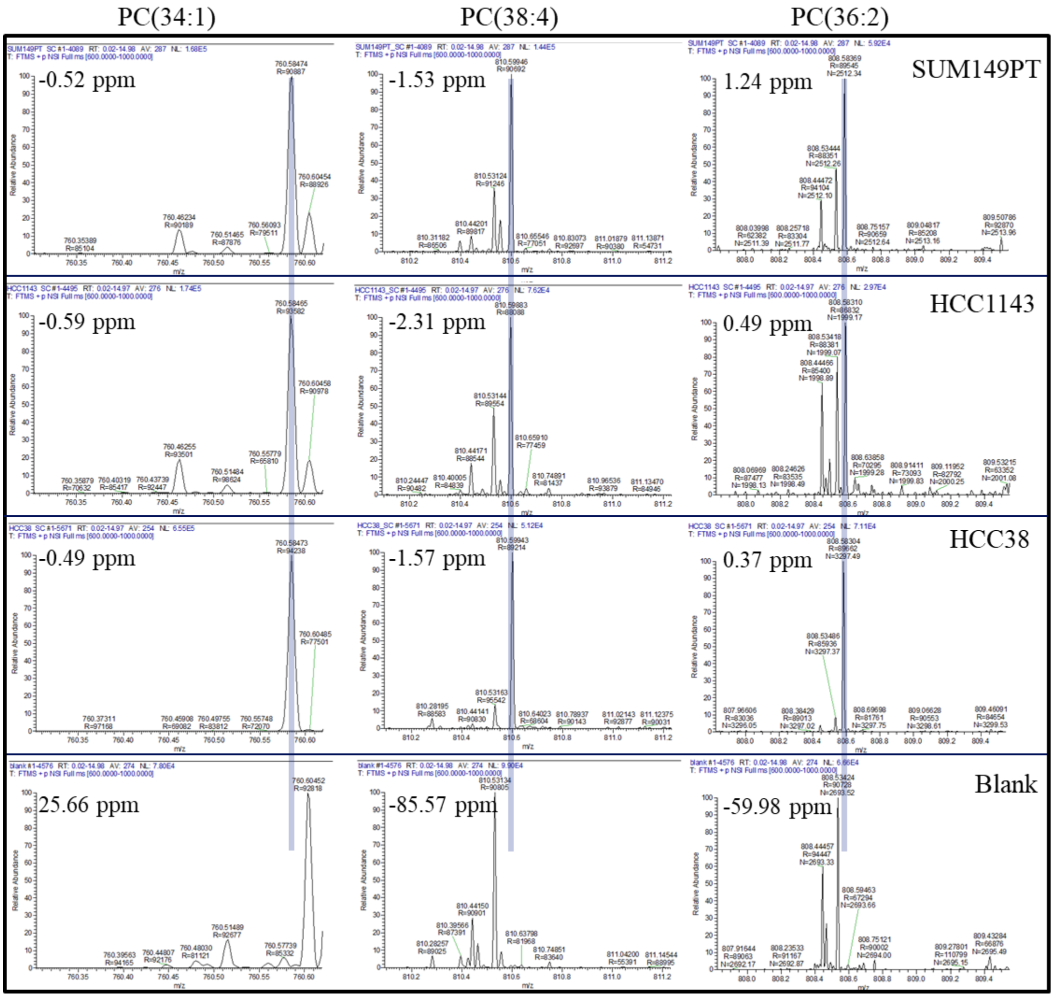

Figure S3. Representative MS[1] spectra of a single cell showing the presence of signals for MS[1] annotated lipids PC(34:1), PC(38:4) and PC(36:2) in SUM149PT, HCC1143, and HCC38 cell lines.

Classified as Internal | Intern

Table S4. Total detected counts of lipids across all single cell samples in positive ion mode.

Total detected counts in positive ion mode		CE	DG	PC	PE	SM	Total
	HCC1143SC	3	25	45	40	6	119
MS1	HCC38SC	3	25	45	40	6	119
	SUM149PTSC	3	23	45	40	6	119
	HCC1143SC	2	0	37	7	1	47
MS2	HCC38SC	2	0	40	9	5	56
	SUM149PTSC	3	1	34	6	3	47

Table S5. Total detected counts of lipids across all single cell samples in negative ion mode.

Total detected counts in negative ion mode		PC	PE	PG	PS	Total
	HCC1143SC	58	41	7	19	125
MS1	HCC38SC	58	41	7	19	125
	SUM149PTSC	58	41	7	19	125
	HCC1143SC	16	9	0	0	25
MS2	HCC38SC	26	10	2	1	39
	SUM149PTSC	20	6	2	1	29

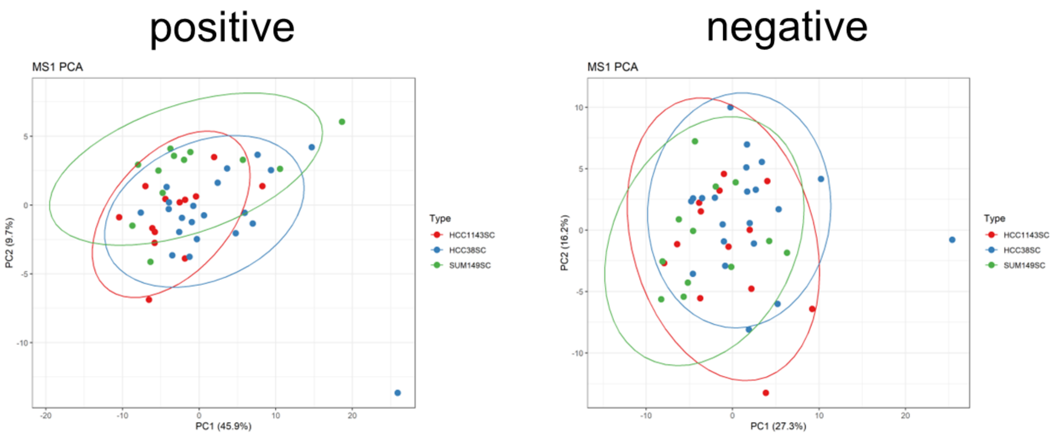

Figure S4. PCA plots of MS1 targets for HCC1143, HCC38, and SUM149PT single cells in positive and negative ionization modes.

Classified as Internal | Intern

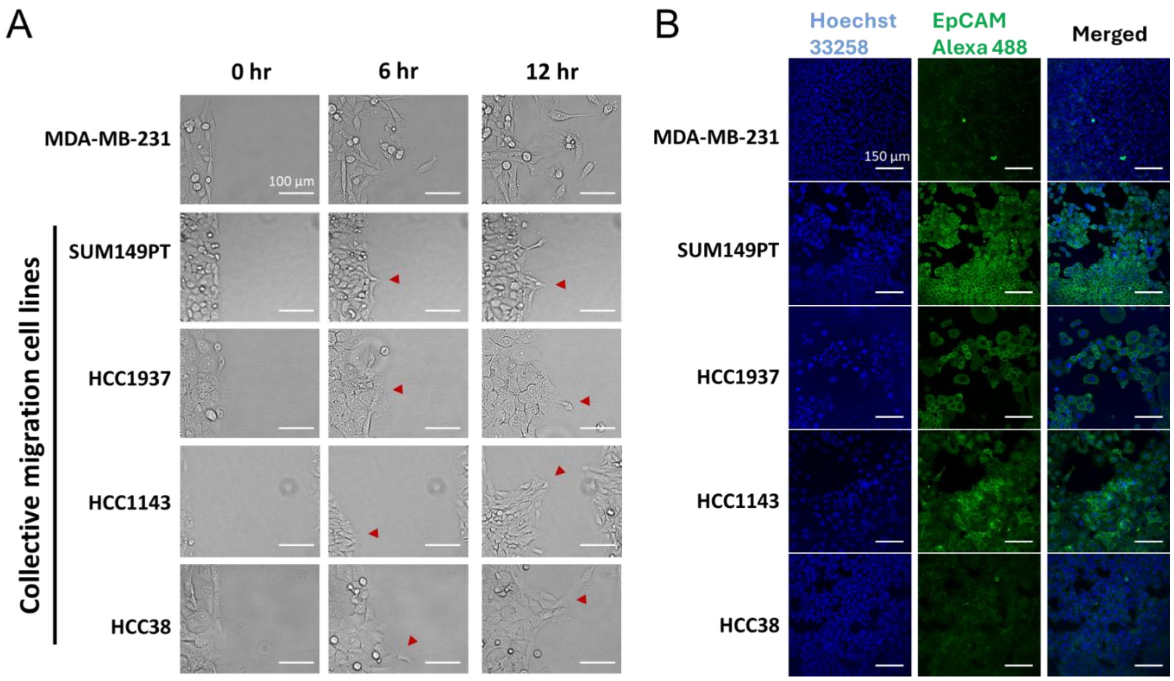

Figure S5. Collective migration behavior and EpCAM expression patterns in five TNBC cell lines. (A) Representative time-lapse images of five TNBC cell lines acquired at 0, 6, and 12 hr. Triangles in red, leader-follower collective chains. Scale bar, 100 µm. (B) Immunofluorescence staining of EpCAM (green, Alexa Fluor 488) and nuclei (blue, Hoechst 33342) in the same cell lines. Scale bar, 150 µm.

Table S6. Fold changes and p-values of significant features that were matched to lysates-derived target list in HCC38 and HCC1143.

Cell line	Molecule name	log2FC	p-value	mode
HCC38	DG(32:2) [M+Na]/ DG(34:5) [M+H]	-4.42	0.01	Positive
	DG(40:6) [M+Na]/DG (42:9) [M+H]	3.85	0.03	Positive
	PE(34:4) [M-H]	0.57	0.03	Negative
	PE(41:6) [M-H]	-3.00	0.02	Negative
HCC1143	PC(34:1) [M+H]	3.29	0.03	Positive
	PS(36:3) [M-H]	-3.07	0.02	Negative
	PC(37:6) [M+HCOO]	-1.72	0.01	Negative

Classified as Internal | Intern

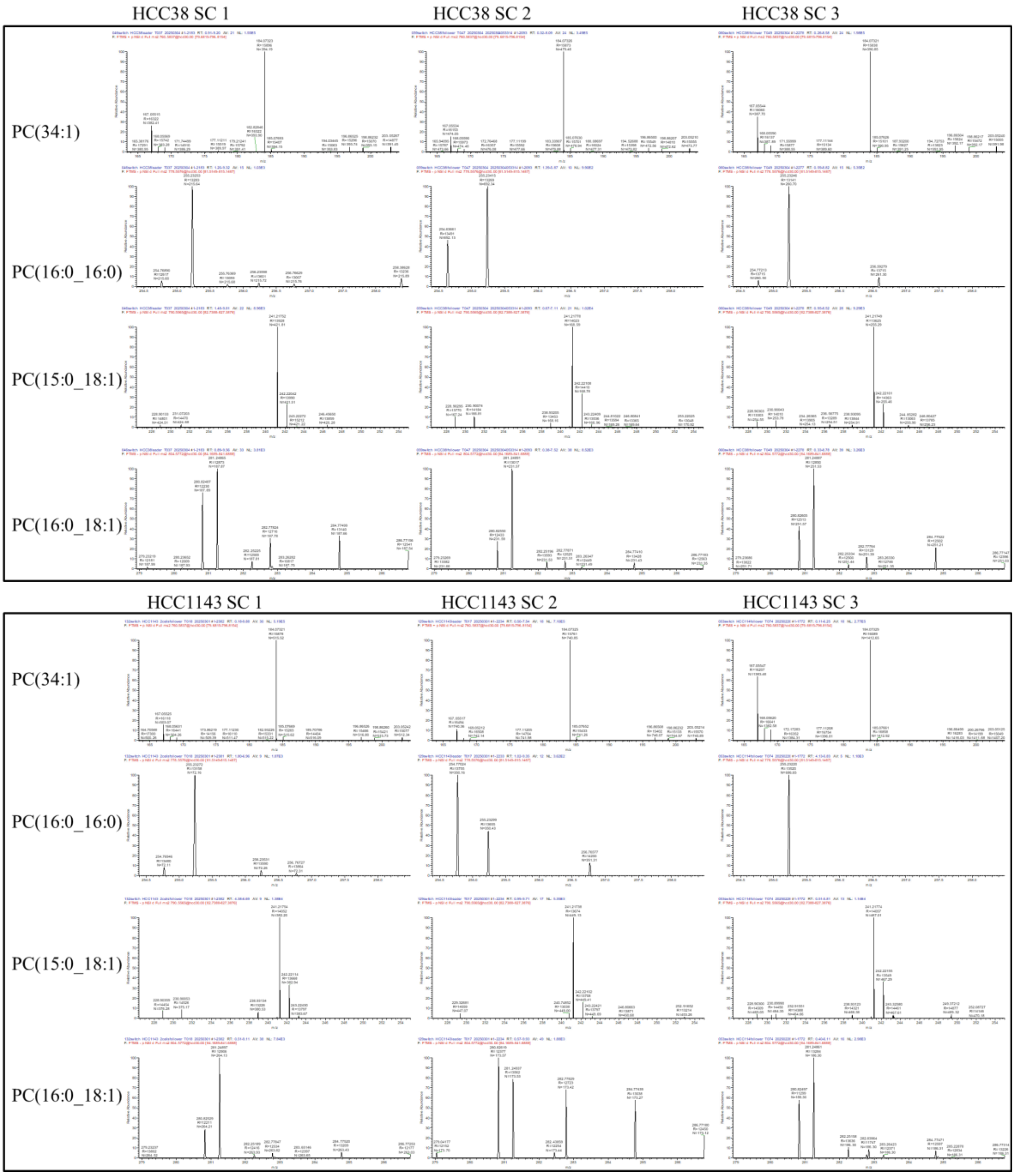

Figure S6. Representative MS/MS spectra of PC species in single cells from HCC38 and HCC1143. Zoomed-in views. Fragment ions corresponding to PC(34:1), PC(16:0_16:0), PC(15:0_18:1), and PC(16:0_18:1) are shown. PC(34:1) displayed the fragment ion at m/z 184.0733 (precursor m/z 760.5837) in positive ion mode. In negative ion mode, fragment ions at m/z 255.2325 (precursor m/z 778.5576), 241.2177 (precursor m/z 790.5565), and 281.2486 (precursor m/z 804.5772), correspond to fatty acyl fragments from PC(16:0_16:0), PC(15:0_18:1), and PC(16:0_18:1), respectively.

Classified as Internal | Intern

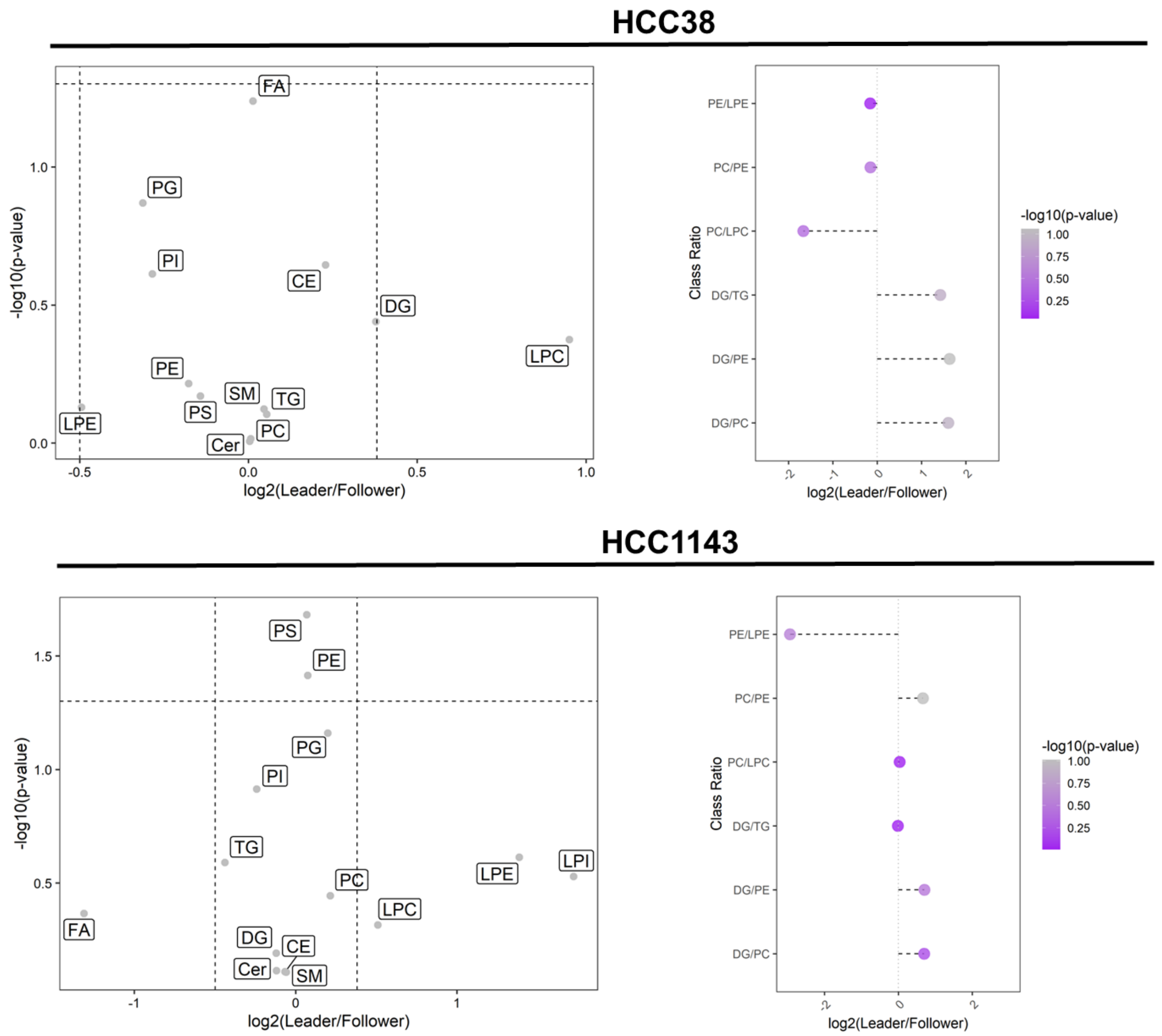

Figure S7. Volcano plots of average expression levels of each lipid class and class ratio alterations between leader and follower cells in HCC38 (above) and HCC1143 (down).

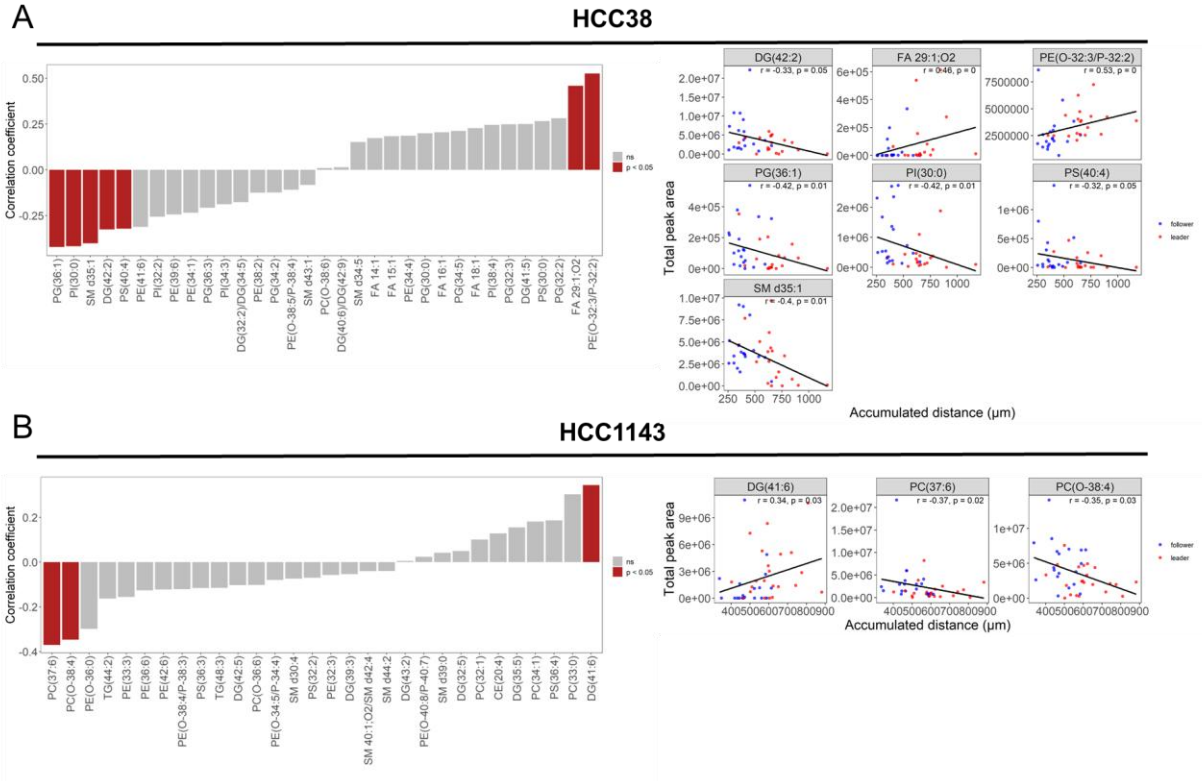

Figure S8. Barplots of the ranked coefficients and scatter plots of highly correlated targets ($p < 0.05$) between individual lipid compounds and the migration accumulated distances of single cells in HCC38 (A) and in HCC1143 (B). The correlation coefficients were calculated using the Spearman method. Columns are highlighted with red hue when *p*-values were less than 0.05.

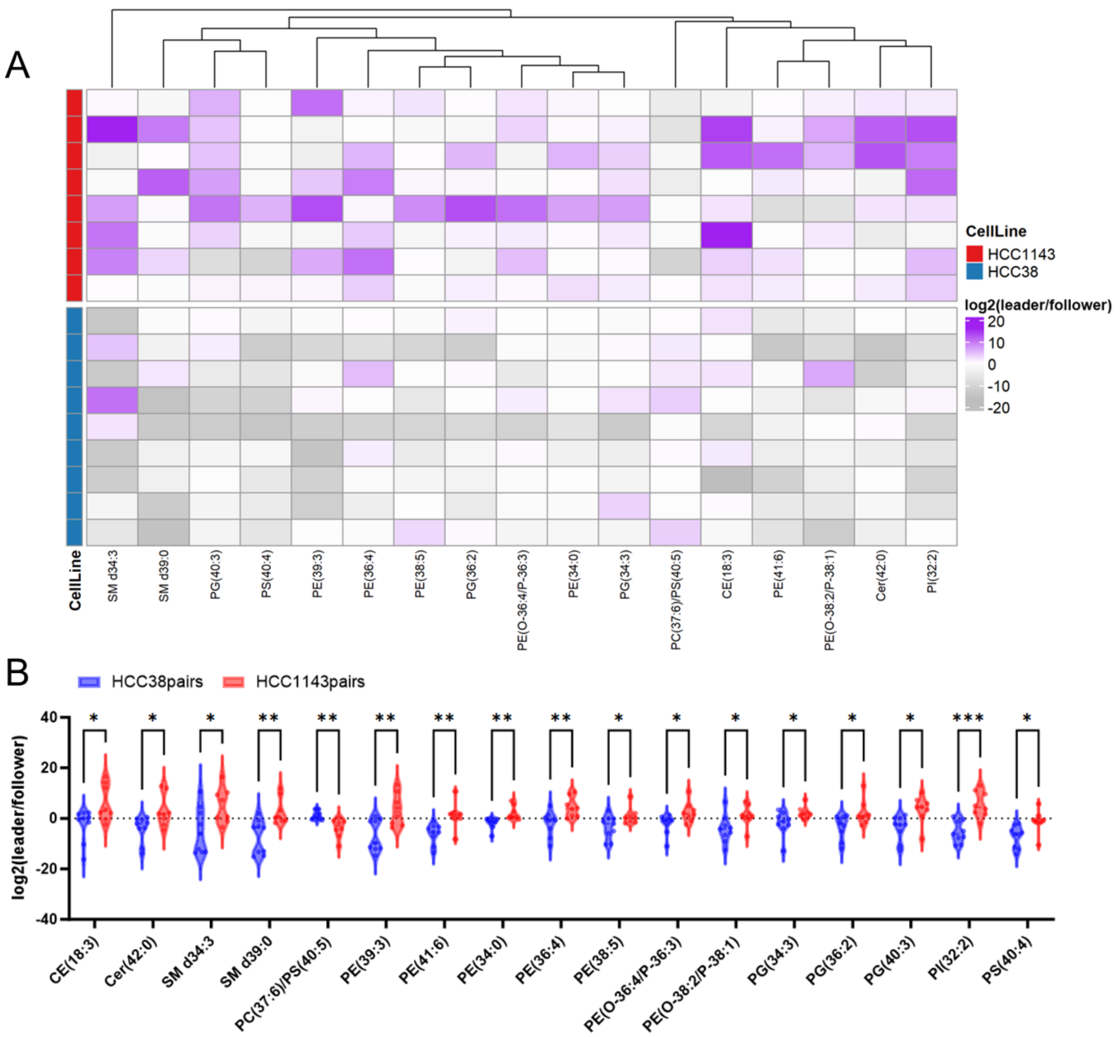

Figure S9. Heatmap (A) and violin plots (B) showed significant leader-to-follower differences between HCC38 and HCC1143 cell lines ($p < 0.05$). For HCC38, leader-follower pairs were n = 9; for HCC1143 pairs, n = 8. For violin plots, data are presented as min to max all points in GraphPad Prism, and analyzed using multiple unpaired *t*-tests (version 10.1.2).

Classified as Internal | Intern
