# Supplementary material for: Imaging‐Guided Live Single‐Cell Lipid Profiling of Leader and Follower Cells During Collective Migration of Triple‐Negative Breast Cancer Cells
Source: Adv Sci (Weinh). 2026 Jun 16:e75862. Online ahead of print. doi: 10.1002/advs.75862 (PMC13336123; doi:10.1002/advs.75862)
Supplement: Supplementary file 2 — Supporting File 2: advs75862‐sup‐0002‐data.zip. [file ADVS-9999-e75862-s002.zip › advs75862-sup-0002-data/Single cell metadata.pdf]

Single cell metadata

Table 1. Single-cell migration tracking data for HCC38 cells.

Sample number	Type	Track number	Accumulated distance (μm)	Velocity (μm/min)
001	Leader	1	N/A	N/A
002	Leader	2	776.067	0.808404
005	Leader	3	902.124	0.939712
007	Leader	4	845.987	0.863252
019	Leader	5	819.599	0.836326
022	Leader	6	853.115	0.870526
025	Leader	7	545.699	0.556836
027	Leader	8	955.361	1.01634
035	Leader	0	953.345	1.0142
037	Leader	9	721.515	0.751578
038	Leader	10	407.092	0.424054
041	Leader	11	515.441	0.536918
043	Leader	12	453.687	0.472591
045	Leader	13	661.714	0.689286
048	Leader	14	352.886	0.367589
056	Leader	15	650.299	0.677395
059	Leader	16	621.571	0.64747
063	Leader	17	753.029	0.768397
064	Leader	18	648.167	0.661395
066	Leader	19	655.104	0.668474
068	Leader	20	1173.89	1.2228
070	Leader	21	630.953	0.657242
074	Leader	22	632.328	0.658674
078	Leader	23	697.866	0.726944
003	Follower	1	395.284	0.411754
009	Follower	2	534.723	0.545636
012	Follower	3	621.486	0.647381
020	Follower	4	654.654	0.668014
021	Follower	5	488.086	0.498047
024	Follower	0	596.832	0.663146
028	Follower	6	304.913	0.324375
030	Follower	7	372.783	0.414203
033	Follower	8	410.842	0.446568
034	Follower	9	257.657	0.268392
039	Follower	10	362.885	0.378005
040	Follower	11	337.526	0.35159
042	Follower	12	263.177	0.274142
044	Follower	13	593.326	0.618048
046	Follower	14	399.801	0.41646
047	Follower	15	352.204	0.374685
049	Follower	16	348.83	0.363365
052	Follower	17	512.918	0.53429

055	Follower	18	393.13	0.409511
057	Follower	19	452.903	0.471774
067	Follower	20	305.384	0.318108
069	Follower	21	407.495	0.424474
073	Follower	22	N/A	N/A

Table 2. Single-cell migration tracking data for HCC1143 cells.

Sample number	Type	Track number	Accumulated distance (μm)	Velocity (μm/min)
096	follower	N/A	N/A	N/A
121	follower	N/A	N/A	N/A
020	follower	1	462.873	0.428586
022	follower	2	445.305	0.397594
036	follower	3	561.469	0.584864
037	follower	4	819.895	0.854057
053	follower	5	418.019	0.444701
056	follower	6	470.641	0.490251
061	follower	7	471.221	0.436315
068	follower	8	523.806	0.476188
074	follower	9	460.005	0.425931
075	follower	10	443.056	0.410237
077	follower	11	613.685	0.568227
079	follower	12	337.078	0.300963
083	follower	13	465.774	0.415869
087	follower	14	523.43	0.467349
099	follower	15	551.029	0.510212
102	follower	16	588.898	0.545276
108	follower	17	588.823	0.545207
111	follower	18	347.6	0.321852
063	follower	19	583.059	0.539869
097	follower	20	432.613	0.400568
013	leader	N/A	N/A	N/A
123	leader	N/A	N/A	N/A
124	leader	N/A	N/A	N/A
008	leader	1	398.002	0.355359
009	leader	2	442.445	0.39504
010	leader	3	622.583	0.555877
012	leader	4	510.823	0.472984
015	leader	5	576.302	0.533613
017	leader	6	774.702	0.717317
019	leader	7	650.806	0.602598
024	leader	8	809.032	0.72235
029	leader	9	501.922	0.464742
030	leader	10	719.992	0.66666
035	leader	11	577.924	0.602004
040	leader	12	618.851	0.644636

048	leader	13	500.079	0.520915
049	leader	14	533.027	0.555237
050	leader	15	564.046	0.587548
051	leader	16	603.611	0.642139
052	leader	17	742.955	0.773911
054	leader	18	597.191	0.622074
055	leader	19	524.331	0.546178
060	leader	20	529.871	0.490622
069	leader	21	603.068	0.548244
073	leader	22	401.959	0.372185
082	leader	23	673.962	0.601752
084	leader	24	662.268	0.591311
088	leader	25	881.756	0.816441
109	leader	26	462.831	0.428548
110	leader	27	592.069	0.548212

Table 3. Metadata of HCC38 single-cell samples.

Sample order	Sample date	Dish No.	Passage No.	Type	sample duration (min)	Measure _order	MS_ Raw File Name
001	2025_02_03	1	3	Leader	22	1	001switch_HCC38leader_T001_20250301.raw
002	2025_02_03	1	3	Leader	28	2	002switch_HCC38leader_T002_20250301.raw
003	2025_02_03	1	3	Follower	38	15	015switch_HCC38follower_T003_20250303.raw
005	2025_02_05	2	4	Leader	20	3	003switch_HCC38leader_T005_20250301.raw
007	2025_02_05	2	4	Leader	54	4	004switch_HCC38leader_T007_20250301_20250301055110.raw
009	2025_02_05	2	4	Follower	70	19	019switch_HCC38follower_T009_20250303.raw
012	2025_02_05	3	4	Follower	21	6	006switch_HCC38leader_T012_20250301_20250301062747.raw
019	2025_02_06	4	5	Leader	17	33	033switch_HCC38Leader_T019_20250303.raw
020	2025_02_06	4	5	Follower	27	24	024switch_HCC38follower_T020_20250303.raw
021	2025_02_06	4	5	Follower	32	25	025switch_HCC38follower_T021_20250303_20250303073500.raw
022	2025_02_06	4	5	Leader	38	34	034switch_HCC38Leader_T022_20250303.raw
024	2025_02_06	5	5	Follower	12	26	026switch_HCC38follower_T024_20250303.raw
025	2025_02_06	5	5	Leader	17	37	037switch_HCC38leader_T025_20250304_20250304095640.raw
027	2025_02_06	5	5	Leader	41	38	038switch_HCC38leader_T027_20250304.raw

028	2025_02_06	5	5	Follower	52	28	028switch_HCC38follower_T028_20250303_20250303083133.raw
030	2025_02_06	5	5	Follower	68	29	029switch_HCC38follower_T030_20250303_20250303084700.raw
033	2025_02_06	5	5	Follower	91	30	030switch_HCC38follower_T033_20250303_20250303090951.raw
034	2025_02_06	5	5	Follower	87	31	031switch_HCC38follower_T034_20250303.raw
035	2025_02_06	5	5	Leader	97	39	039switch_HCC38leader_T035_20250304.raw
037	2025_02_07	6	6	Leader	26	46	046switch_HCC38leader_T037_20250304.raw
038	2025_02_07	6	6	Leader	38	47	047switch_HCC38leader_T038_20250304.raw
039	2025_02_07	6	6	Follower	44	54	054switch_HCC38lfollower_T039_20250304.raw
040	2025_02_07	6	6	Follower	50	55	055switch_HCC38lfollower_T040_20250304_20250304044045.raw
041	2025_02_07	6	6	Leader	67	48	048switch_HCC38leader_T041_20250304_20250304013231.raw
042	2025_02_07	6	6	Follower	72	56	056switch_HCC38lfollower_T042_20250304_20250304043157.raw
043	2025_02_07	6	6	Leader	81	49	049switch_HCC38leader_T043_20250304.raw
044	2025_02_07	6	6	Follower	90	57	057switch_HCC38lfollower_T044_20250304_20250304050448.raw
045	2025_02_07	6	6	Leader	98	50	050switch_HCC38leader_T045_20250304.raw
046	2025_02_07	6	6	Follower	103	58	058switch_HCC38lfollower_T046_20250304_20250304054452.raw
047	2025_02_07	7	6	Follower	11	59	059switch_HCC38lfollower_T047_20250304_20250304053314.raw
048	2025_02_07	7	6	Leader	25	51	051switch_HCC38leader_T048_20250304.raw
049	2025_02_07	7	6	Follower	30	60	060switch_HCC38lfollower_T049_20250304.raw
052	2025_02_07	7	6	Follower	56	61	061switch_HCC38lfollower_T052_20250304.raw
055	2025_02_07	7	6	Follower	88	40	040switch_HCC38follower_T055_20250304_20250304110632.raw
056	2025_02_07	7	6	Leader	94	43	043switch_HCC38leader_T056_20250304.raw
057	2025_02_07	7	6	Follower	104	41	041switch_HCC38follower_T057_20250304.raw
059	2025_02_07	7	6	Leader	118	44	044switch_HCC38leader_T059_20250304.raw

063	2025_02_08	8	7	Leader	29	65	065switch_HCC38leader_T063_20250304_20250304071006.raw
064	2025_02_08	8	7	Leader	40	66	066switch_HCC38leader_T064_20250304.raw
066	2025_02_08	8	7	Leader	62	67	067switch_HCC38leader_T066_20250304.raw
067	2025_02_08	9	7	Follower	16	76	076switch_HCC38follower_T067_20250304.raw
068	2025_02_08	9	7	Leader	22	68	068switch_HCC38leader_T068_20250304.raw
069	2025_02_08	9	7	Follower	25	77	077switch_HCC38follower_T069_20250304_20250304092243.raw
070	2025_02_08	9	7	Leader	35	69	069switch_HCC38leader_T070_20250304.raw
073	2025_02_08	9	7	Follower	57	80	080switch_HCC38follower_T073_20250304.raw
074	2025_02_08	9	7	Leader	73	70	070switch_HCC38leader_T074_20250304.raw
078	2025_02_08	9	7	Leader	97	73	073switch_HCC38leader_T078_20250304.raw

Table 4. Metadata of HCC1143 single-cell samples.

Sample order	Sample date	Dish No.	Passage No.	Type	sample duration (min)	Measure _order	MS_ Raw File Name
008	2025_01_14	1	3	leader	45	118	118switch_HCC1143leader_T008_20250228_5um.raw
009	2025_01_14	1	3	leader	55	119	119switch_HCC1143leader_T009_20250228_5um.raw
010	2025_01_14	1	3	leader	75	120	120switch_HCC1143leader_T010_20250228.raw
012	2025_01_14	2	4	leader	52	121	121switch_HCC1143leader_T012_20250228.raw
013	2025_01_14	2	4	leader	63	122	122switch_HCC1143leader_T013_20250228_20250228090055.raw
015	2025_01_14	2	4	leader	82	127	127switch_HCC1143leader_T015_20250301_20250301125337.raw
017	2025_01_14	2	4	leader	104	129	129switch_HCC1143leader_T017_20250301.raw
019	2025_01_14	2	4	leader	117	130	130switch_HCC1143leader_T019_20250301.raw
020	2025_01_14	2	4	follower	128	131	131switch_HCC1143follower_T020_20250301.raw
022	2025_01_15	3	5	follower	40	114	114switch_HCC1143follower_022_20250228_20250228071315.raw
024	2025_01_15	3	5	leader	82	105	105switch_HCC1143Leader_T024_20250228.raw
029	2025_01_15	4	6	leader	77	108	108switch_HCC1143leader_T029_20250228.raw

030	2025_01_15	4	6	leader	83	109	109switch_HCC1143leader_T030_20250228.raw
035	2025_01_16	5	7	leader	58	32	032switch_HCC1143Leader_T035_20250225.raw
036	2025_01_16	5	7	follower	62	34	034switch_HCC1143Follower_T036_20250225.raw
037	2025_01_16	5	7	follower	76	35	035switch_HCC1143Follower_T037_20250225.raw
040	2025_01_16	5	7	leader	105	33	033switch_HCC1143Leader_T040_20250225_20250225043133.raw
048	2025_01_21	6	8	leader	27	36	036switch_HCC1143Leader_T048_20250225.raw
049	2025_01_21	6	8	leader	34	37	037switch_HCC1143Leader_T049_20250225.raw
050	2025_01_21	6	8	leader	40	38	038switch_HCC1143Leader_T050_20250225_20250225054208.raw
051	2025_01_21	6	8	leader	56	39	039switch_HCC1143Leader_T051_20250225.raw
052	2025_01_21	6	8	leader	69	40	040switch_HCC1143Leader_T052_20250225.raw
053	2025_01_21	6	8	follower	76	41	041switch_HCC1143Follower_T053_20250225.raw
054	2025_01_21	6	8	leader	85	42	042switch_HCC1143Leader_T054_20250225_20250225063334.raw
055	2025_01_21	6	8	leader	90	43	043switch_HCC1143Leader_T055_20250225.raw
056	2025_01_21	6	8	follower	103	61	061switch_HCC1143Follower_T056_20250226.raw
060	2025_01_21	7	9	leader	47	44	044switch_HCC1143Leader_T060_20250225.raw
061	2025_01_21	7	9	follower	62	62	062switch_HCC1143Follower_T061_20250226.raw
063	2025_01_21	7	9	follower	93	63	063switch_HCC1143Follower_T063_20250226.raw
068	2025_01_23	8	10	follower	47	52	052switch_HCC114follower_T068_20250226_20250226105738.raw
069	2025_01_23	8	10	leader	53	49	049switch_HCC1143leader_T069_20250226.raw
073	2025_01_23	9	11	leader	34	50	050switch_HCC1143leader_T073_20250226.raw
074	2025_01_23	9	11	follower	39	53	053switch_HCC114follower_T074_20250226.raw
075	2025_01_23	9	11	follower	45	54	054switch_HCC114follower_T075_20250226.raw
077	2025_01_23	9	11	follower	84	55	055switch_HCC114follower_T077_20250226.raw
079	2025_01_25	10	12	follower	10	86	086switch_HCC1143follower_T079_20250227.raw
082	2025_01_25	10	12	leader	35	80	080switch_HCC1143Leader_T082_20250227.raw
083	2025_01_25	10	12	follower	41	83	083switch_HCC1143follower_T083_20250227.raw

084	2025_01_25	10	12	leader	52	81	081switch_HCC1143Leader_T084_20250227.raw
087	2025_01_25	10	12	follower	75	85	085switch_HCC1143follower_T087_20250227.raw
088	2025_01_25	11	13	leader	19	89	089switch_HCC1143leader_T088_20250227.raw
096	2025_01_27	12	14	follower	29	71	071switch_HCC1143follower_T096_20250227_20250227105602.raw
097	2025_01_27	13	15	follower	33	72	072switch_HCC1143follower_T097_20250227.raw
099	2025_01_27	13	15	follower	53	74	074switch_HCC1143follower_T099_20250227.raw
102	2025_01_27	13	15	follower	86	75	075switch_HCC1143follower_T102_20250227.raw
108	2025_01_27	14	16	follower	80	76	076switch_HCC1143follower_T108_20250227.raw
109	2025_01_27	14	16	leader	87	78	078switch_HCC1143Leader_T109_20250227.raw
110	2025_01_27	14	16	leader	102	79	079switch_HCC1143Leader_T110_20250227_20250227015607.raw
111	2025_01_27	14	16	follower	109	77	077switch_HCC1143follower_T111_20250227.raw
121	2025_01_28	15	17	follower	46	100	100switch_HCC1143follower_T121_20250228_20250228111339.raw
123	2025_01_28	15	17	leader	71	22	022switch_HCC1143leader_T123_20250224_20250224021448.raw
124	2025_01_28	15	17	leader	81	102	102switch_HCC1143leader_T124_20250228.raw
